# Nanoparticles in liposomes: a platform for increased antibiotic selectivity in multidrug resistant bacteria in respiratory tract infections

**DOI:** 10.1007/s13346-024-01662-2

**Published:** 2024-07-24

**Authors:** Nathalie E. Fakhoury, Samar Mansour, Mohammad Abdel-Halim, Mostafa M. Hamed, Martin Empting, Annette Boese, Brigitta Loretz, Claus-Michael Lehr, Salma N. Tammam

**Affiliations:** 1 Department of Pharmaceutical Technology, Faculty of Pharmacy & Biotechnology, the German University in Cairo, Cairo, Egypt; 2 Department of Pharmaceutical Chemistry, Faculty of Pharmacy & Biotechnology, the German University in Cairo, Cairo, Egypt; 3 Helmholtz Institute for Pharmaceutical Research Saarland (HIPS), Helmholtz Center for Infection Research, 66123 Saarbrücken, Germany; 4 Department of Pharmacy, Saarland University, 66123 Saarbrücken, Germany

**Keywords:** Nanoparticles in liposomes, *Pseudomonas aeruginosa* biofilms, Antibiotic, Quorum sensing inhibitor, Selectivity

## Abstract

Antibiotic resistance is a cause of serious illness and death, originating often from insufficient permeability into gram-negative bacteria. Nanoparticles (NP) can increase antibiotic delivery in bacterial cells, however, may as well increase internalization in mammalian cells and toxicity. In this work, NP in liposome (NP-Lip) formulations were used to enhance the selectivity of the antibiotics (3C and tobramycin) and quorum sensing inhibitor (HIPS-1635) towards *Pseudomonas aeruginosa* by fusing with bacterial outer membranes and reducing uptake in mammalian cells due to their larger size. Poly (lactic-co-glycolic) acid NPs were prepared using emulsion solvent evaporation and incorporated in larger liposomes. Cytotoxicity and uptake studies were conducted on two lung cell lines, Calu-3 and H460. NP-Lip showed lower toxicity and uptake in both cell lines. Then formulations were investigated for suitability for oral inhalation. The deposition of NP and NP-Lip in the lungs was assessed by next generation impactor and corresponded to 75% and 45% deposition in the terminal bronchi and the alveoli respectively. Colloidal stability and mucus-interaction studies were conducted. NP-Lip showed higher diffusion through mucus compared to NPs with the use of nanoparticle tracking analyzer. Moreover, the permeation of delivery systems across a liquid-liquid interface epithelial barrier model of Calu-3 cells indicated that NP-Lip could cause less systemic toxicity upon in-vivo like administration by aerosol deposition. Monoculture and *Pseudomonas aeruginosa* biofilm with Calu-3 cells co-culture experiments were conducted, NP-Lip achieved highest toxicity towards bacterial biofilms and least toxicity % of the Calu-3 cells. Therefore, the NP- liposomal platform offers a promising approach for enhancing antibiotic selectivity and treating pulmonary infections.

**Graphical abstract:**

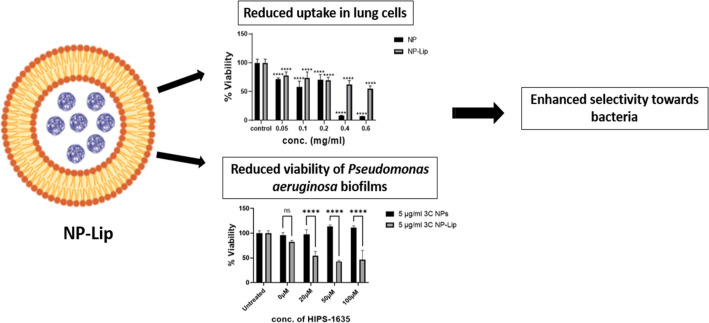

**Supplementary Information:**

The online version contains supplementary material available at 10.1007/s13346-024-01662-2.

## Introduction

Bacterial drug resistance is one of the leading causes of death and morbidity in the world. It has been and will continue to threaten the lives of many people globally [1]. It also presents a heavy economic burden on healthcare systems [1]. Bacteria have various mechanisms by which they can resist the action of drugs. They use drug efflux pumps to expel antibiotics out of their cells resulting in the ineffectiveness of the medications [2]. Additionally, the complex structure of bacteria, particularly in gram-negative bacteria, presents a difficulty for drug permeability due to the additional outer membrane composed of lipopolysaccharides [3]. Moreover, some bacteria are also capable of biofilm formation, which shields the bacteria from drugs and provides an additional barrier for drug entry in bacterial cells [4].

*Pseudomonas aeruginosa (P. aeruginosa)* is a bacteria of interest in this work since it is a gram-negative bacteria and famous former of biofilms, consequently leading to severe lung infections in immunocompromised and cystic fibrosis patients [5]. While the discovery of novel antimicrobial agents is essential to eradicate the resistant bacteria [6], most newly developed compounds exhibit poor drug solubility and plasma stability [7]. By the use of nanotechnology we tackle the issues encountered with the newly developed compounds [8–10], and for that reason in previous work, nanoparticle in liposome (NP-Lip) delivery system was used to encapsulate antibiotics for the treatment of different strains of *E. coli* and *P. aeruginosa* in their planktonic form [11]*.* The success of this delivery system was attributed to the presence of liposomes which have the ability to fuse with the outer membrane of bacterial cells allowing the release of drug loaded nanoparticles (NP) [12]. Since drugs are loaded in NPs the drug is shielded from the drug efflux pumps present in the periplasm and is able to exert its mechanism in the cytosol [13]. Furthermore, the NP-Lip is considered to be less toxic due to the reduced uptake in mammalian cells owing to its larger size relative to the small NPs [14], therefore allowing higher selectivity towards bacteria.

In this work, we focus on the use of drug loaded NP-Lip for the treatment of *P. aeruginosa* biofilms, a more resistant form in the lungs [15]. The bactericidal efficiency of our delivery system was evaluated using presto blue assay in monocultures and using colony forming units/ml in *P. aeruginosa* biofilm and Calu-3 cells co-culture model. NP-Lip were loaded with novel antimicrobial agents, 3C and HIPS-1635 and the standard antibiotic tobramycin. 3C, a 5-cyanothiazolyl urea derivative is a newly developed antibiotic that acts as a Mur A/ Mur B inhibitor [16], used for the inhibition of cell walls of bacterial cells and proven effective against multiple strains of gram-negative bacteria [11]. Furthermore, HIPS-1635, an anti-biofilm compound acts as a Pqsr inverse agonist inhibiting the quorum sensing system of *P. aeruginosa* [17]. Both antibiotics (3C and tobramycin) were loaded in the NPs while the HIPS-1635 was loaded in Lip. Therefore, the dual encapsulation of antibiotics with quorum sensing inhibitors in our delivery system offers a quite promising approach for the selective treatment of *P. aeruginosa* biofilms in the lungs.

## Material and methods

### Materials

PLGA (ratio 50/50 Mwt:17000 g/mol, source: Changchun Foliaplast Bio-Tech c., Ltd.,China), Cholesterol (source: Techno Pharmchem, India) and Soybean lecithin (source: Beijing Yuan Hua Mei Lecithin Sci-Tech Co., Ltd., China) were purchased.

Tobramycin (Sigma Aldrich, Germany), *P. aeruginosa* (PAO1, ATCC) and Calu-3 cells (ATCC) were utilized. 3C was synthesized in the Germany University in Cairo. HIPS-1635 was synthesized at Helmholtz Institute for Pharmaceutical Research Saarland and kindly provided by the department of Drug Design and Optimization.

### Methods

#### Formulation and characterization of nanoparticles, liposomes and nanoparticles in liposomes

##### Nanoparticles

Polymeric NPs were prepared by the emulsion solvent evaporation method as detailed in earlier work [11]. Briefly, 2 mg of poly (lactic co-glycolic acid) (PLGA) were dissolved in 2 ml ethyl acetate and added dropwise to 10 ml of 1% (v/v) Tween 80 with continuous stirring at 800 rpm. The resultant emulsion was sonicated at 45% for 10 minutes on ice followed by stirring overnight to allow the evaporation of ethyl acetate. NPs were concentrated and purified using 100 kDa Amicon® ultra-4 centrifugal filter units.

##### Liposomes

Liposomes (Lip) were formulated by the dispersion of lipids in ultrapure water as detailed in earlier work [11]. Briefly, 250 μl of dimethyl sulfoxide (DMSO) were added to 2 mg of cholesterol and sonicated in a water-bath sonicator for 10 seconds. Then, 16 mg soybean lecithin and 5 ml of ultrapure water were added and vortexed for 6 minutes at 2500 rpm. Lip were collected through centrifugation at 8000 rpm for 10 minutes at 10 °C [11], and the pellet was reconstituted in 1 ml ultrapure water.

##### Nanoparticles in liposomes

NPs were formulated as previously described and concentrated by adding 5 ml of formulation to the 100 kDa Amicon® ultra-4 centrifugal filter units and subjecting them to centrifugation until 500 μl of NP suspension was obtained. This concentrated NP suspension corresponded to 2 mg/ml in terms of PLGA. Concentrated particles were then added to the 16 mg soybean lecithin and 250 μl of DMSO containing cholesterol in 5 ml ultrapure water and were all mixed together by vortex agitation at 2500 rpm for 6 minutes.

NP, Lip and NP-Lip hydrodynamic diameter (HD) and zeta potential (ZP) were determined by a Malvern Zetasizer using three independent batches each analyzed in triplicates and results were expressed as mean and error as standard deviation (SD). Particle morphology was also determined by scanning and transmission electron microscopy (SEM and TEM respectively). To prepare particles for TEM, one drop of each formulation was placed on a carbon-coated film 300 mesh copper grid followed by staining with 1 M uranyl acetate solution. JEOL-JEM 1010 Transmission Electron Microscopy (TEM; JEOL Ltd., Tokyo, Japan) was used to examine the samples. For SEM analysis, particles were first sputtered with gold for 2 minutes at 15 mA using Anatech Hummer 8.0 sputter system, followed by assessment using a LEO Supra 55 field emission scanning electron microscope (SEM; Zeiss, Oberkochen, Germany).

#### Determination of NP in Lip incorporation

NPs were initially loaded with rhodamine B fluorescent dye and prepared as previously described using the emulsion solvent evaporation method. Then fluorescent NPs were concentrated and purified using 100 kDa Amicon® ultra-4 centrifugal filter units and incorporated into Lip as detailed in the previous section yielding NP-Lip. NP-Lip were centrifuged at 8000 rpm for 10 minutes at 10 °C and supernatant was collected. Fluorescence intensity was then determined for an aliquot of 100 μl of the supernatant at excitation/emission wavelength of 546/568 nm using a plate reader. Concentration of the unincorporated NPs was determined by preparing calibration curves of fluorescent NPs (0.001–0.2 mg/ml). NP incorporation (I%) in Lip was then determined indirectly as detailed below.

$$\textrm{I}\%=\frac{\left(\textrm{Concentration}\ \textrm{of}\ \textrm{NPs}\ \textrm{added}\hbox{--} \textrm{Concentration}\ \textrm{of}\ \textrm{unincorporated}\ \textrm{NPs}\right)}{\textrm{Concentration}\ \textrm{of}\ \textrm{NPs}\ \textrm{added}}\textrm{X}100$$

I% was determined from three independently prepared batches of NP-Lip and results were expressed mean I% and error as SD.

To further confirm NP encapsulation, NP, Lip and NP-Lip were assessed by confocal laser scanning microscopy (CLSM; Leica, TCS SP8). Rhodamine B loaded NPs were prepared as detailed earlier. Fluorescent Lip were prepared using TopFluor® cholesterol (Avanti Lipid, USA). To do so, Lip were prepared as detailed earlier, however 2% of their cholesterol content was replaced with TopFluor cholesterol. NP-Lip were prepared using fluorescent NPs and TopFluor cholesterol. NPs were imaged with emission filter set to 568 nm excited by a laser line at 546 nm. Lip were imaged with emission filter set to 507 nm excited by a laser line at 495 nm. Resolution was set to 1024 × 1024 pixels and the scan speed to 200 Hz. NP, Lip and NP-Lip images were enhanced using Imaris software as detailed in supplementary file 1, 2 and 3 respectively.

#### In vitro evaluation of NP and NP-Lip cell cytotoxicity and cell uptake

##### Cytotoxicity

The 3-(4, 5-dimethyl-2-thiazolyl)-2, 5-diphenyltetrazolium bromide assay (MTT) was used to assess cytotoxicity of the formulations. In more detail, Calu-3 and H460 cells were seeded in a 96 well plate at a density of 5× 10^4^ and 2× 10^4^ cells/well respectively and allowed to adhere overnight. The medium from both cells lines was aspirated and both were treated with increasing concentrations of NPs and NP-Lip (0.05, 0.1, 0.2, 0.4 and 0.6 mg/ml in terms of PLGA content). Cells were incubated with the formulations for 4 and 24 hours. Then medium was aspirated and MTT solution with a concentration of 0.5 mg/ml was added to the cells and incubated for 3 hours. MTT solution was aspirated followed by the addition of DMSO and absorbance at a wavelength of 570 nm was measured by plate reader against a control (untreated cells).

##### Cell uptake

Calu-3 and H460 cells were seeded at a density of 5× 10^4^ and 2× 10^4^ respectively in a 96 well plate and were allowed to adhere overnight. On the following day, the medium was aspirated and cells were treated with fluorescent NPs and NP-Lips for 4 hours. Calu-3 cells were treated with concentrations of 0.2 and 0.4 mg/ml while H460 cells were treated with 0.05, 0.1 and 0.2 mg/ml (in terms of PLGA content). After incubation, the formulation containing media was aspirated and cells were washed twice with PBS and finally fluorescence intensity was determined at excitation/emission wavelength of 546/568 nm using a plate reader. Calibration curves constructed using serial dilution of fluorescent NPs and NP-Lips were used to determine the concentrations as detailed in [18]. Results were expressed as NP or NP-Lip concentration associated to cells and error as SD.

#### Evaluation of the suitability of NP-Lip for pulmonary drug delivery

##### NP and NP-Lip nebulization and evaluation of lung deposition pattern by next generation impactor (NGI)

Fluorescent NPs were prepared as detailed earlier, NP-Lip were prepared with fluorescent NPs, but not with TopFluor cholesterol. An Aeroneb lab nebulizer with a volume mean diameter (VMD) of 2.5–4 was used to nebulize 2 ml of both formulations at a flow rate of 15 and 30 L/min. Nebulization time was adjusted to 15 minutes and particles were conveyed through the NGI, where they were impacted on eight consecutive stages based on a well-characterized aerodynamic size cut-offs. After actuation, the contents of the nebulizer, mouthpiece adaptor, induction port, pre-separators, stages 1–7 and the micro-orifice contactor (MOC) were washed with deionized water. Rhodamine was used to assess the deposition pattern by measuring the fluorescence intensities of the solutions by plate reader at 546 nm excitation filter and emission at 568 nm to determine the amount of powder retained on each stage of the NGI. Then the median mass aerodynamic diameters (MMAD) were generated for both NP and NP-Lip. After nebulization the size of particles was measured using Malvern Zetasizer.

##### Evaluation of NP and NP-Lip behavior in mucus surrogate

Artificial mucus was prepared as detailed in [19]. Fluorescent NPs were prepared as detailed earlier, NP-Lip were prepared with fluorescent NPs, but not with TopFluor cholesterol. To 1 ml of the prepared mucus, 40 μl of NPs and NP-Lip were added respectively followed by mixing by vortex agitation. The NP in mucus and NP-Lip in mucus were diluted 5000 and 3000 times respectively in PBS. Then samples were injected in the nanoparticle tracking analyzer (Nanosight LM-10, United Kingdom) and particle size and concentration were recorded. Diffusion coefficient of particles in different medium was calculated as detailed in [20].

##### Permeation of NPs and NP-Lip across epithelial barrier

Calu-3 cells were seeded on 24 transwell inserts at a density of 3× 10^4^ cell per insert as detailed in [21]. Cells were allowed to grow in a monolayer for 10–12 days. Transepithelial electrical resistance (TEER) measurements were conducted to ensure tight barrier formation. Cells were washed twice with Krebs-Ringer Bicarbonate (KRB) buffer then left to incubate for 1 hour. TEER was measured again and then cells were treated with fluorescent NPs (0.2 mg/ml final concentration in terms of PLGA), NP-Lip (0.2 mg/ml final concentration in terms of PLGA) and free rhodamine dye (0.02 mg/ml final concentration in terms of rhodamine B) apically. At predetermined time intervals (0, 1, 2, 4, 6 and 24 hours), 100 μl samples were withdrawn, and buffer was used to replenish the withdrawn volume. Fluorescence intensity of samples was measured at excitation/emission wavelength of 546/568 nm using a plate reader. Calibration curves were constructed to determine the concentration of permeated particles.

#### Drug loading and evaluation of encapsulation efficiency

A stock of 1 mg/ml 3C in DMSO was prepared. To load 3C, NPs were prepared as detailed earlier, however 200 μl of 3C stock solution was added to the organic phase during NP formulation. In a similar manner tobramycin (Tbr) was dissolved in methanol (2 mg/ml). To load Tbr, NPs were prepared as detailed earlier, however 200 μl of Tbr stock solution was added to the organic phase during formulation.

For encapsulation efficiency (EE%) determination, 4 ml of 3C NPs and 500 μl of DMSO were added to 100 kDa Amicon® ultra-4 centrifugal filter units and centrifugation at 4000 rpm yielding 500 μl of concentrated NPs. The flow through was collected for quantification of the unencapsulated 3C, using ultra high liquid chromatography – tandem mass spectrometry (UHPLC-MS-MS) as detailed in the supplementary file. In a similar manner Tbr NPs were also centrifuged in 100 kDa Amicon® ultra-4 centrifugal filter units at 4000 rpm yielding 500 μl of concentrated NPs. The flow through was collected for quantification of the unencapsulated Tbr using UHPLC-MS-MS. In both cases calibration curves of either 3C or Tbr were constructed in NP flow through and were used to compute unencapsulated concentration.

To load HIPS-1635 into NP-Lip, a stock of 2 mg/ml of HIPS-1635 in DMSO was prepared, NP-Lip were prepared as detailed earlier, however 500 μl of HIPS-1635 stock solution was added to 4.5 ml of deionized water in which cholesterol and soybean lecithin were dispersed. NP-Lip containing HIPS-1635 were collected through centrifugation at 8000 rpm for 10 minutes at 10 °C. Subsequently, 4 ml of supernatants were collected and added to 100 kDa Amicon® ultra-4 centrifugal filter units and centrifuged at 4000 rpm for 15 minutes. The flow through was collected and used to quantify the concentration of unencapsulated HIPS-1635 using UHPLC-MS-MS as detailed in the supplementary file. A calibration curve of HIPS-1635 in NP-Lip supernatant was constructed to determine unencapsulated concentration.

To ensure that the size and morphology of particles did not change upon drug loading, the size of drug loaded particles was assessed using Malvern Zetasizer and the morphology was assessed using TEM.

#### Evaluation of the ability of NP-Lip to increase antibiotic effectiveness and selectivity in *P. aeruginosa* biofilms

*P. aeruginosa* biofilms were grown as detailed in [22]. Briefly, *P. aeruginosa* with an optical density (OD600) of 0.01 were grown in 96 well plates for 72 hours in a 37 °C incubator and supplemented with M63 medium. Biofilm formation was confirmed by SEM (Evo HD 15, Germany). Biofilm viability was assessed with the following samples: For the free drugs 3C, Tbr as antibiotics and the quorum sensing inhibitor HIPS-1635, 2 concentrations reflecting the 1x and 2x MIC for Tbr, and 0.5x and 1x MIC for 3C were chosen. Same concentrations of antibiotics were encapsulated in NPs and were tested either alone (3C NP and Tbr NP) or in combination with free HIPS-1635 (3C NP + HIPS-1635 and Tbr NP + HIPS-1635). NP-Lip contained either only antibiotic-loaded NP (3C NP-Lip and Tbr NP-Lip), in combination with free HIPS-1635 (3C NP-Lip + HIPS-1635 and Tbr NP-Lip + HIPS-1635) or encapsulation of HIPS-1635 in the Lip (3C NP- H1635-Lip and Tbr NP- H1635-Lip). All treatment groups and concentrations tested are detailed in Table A in the supplementary materials. After 24 hours of incubation, presto blue assay was utilized and absorbance was measured at 570 and 600 nm to assess the viability of biofilms [23].

#### Assessing the cytotoxicity of drug loaded NPs and NP-Lip towards Calu-3 cells

Calu-3 cells were seeded with a density of 5 × 10^4^ cells/well and allowed to adhere overnight. Cells were treated with the same treatment groups and concentrations as those used for the treatment of bacterial biofilms as detailed in Table A in supplementary materials. After 24 hours, cell culture media was aspirated and 100 μl of MTT was added with a concentration of 0.5 mg/ml. Plates were incubated for 3 hours, followed by removal of the of MTT solution and DMSO was added and absorbance was measured at 570 nm using a plate reader.

#### Co-culturing Calu-3 cells with *P. aeruginosa* biofilm

An infection model that was developed by Horstmann et al. was utilized which transfers pre grown biofilms on epithelial cell monolayer [24]. Calu-3 cells were seeded at a density of 1 × 10^5^ cell per well in twelve well inserts and grown into a monolayer for 10 days. Biofilms were grown separately in 24 well plates as previously described 72 hours prior to the start of experiment. On the day of the experiment, medium was aspirated from the lower compartment followed by addition of 0.5 ml of medium supplemented with 0.4% arginine in all wells expect dead control wells to preserve the integrity of monolayers [25]. For dead control wells 0.5 ml of medium supplemented with arginine and 1% triton-X were added basolaterally. Biofilms supernatants were removed and washed twice with KRB buffer before placement apically on the Calu-3 monolayer. As non-infected control KRB buffer was added. After 1 hour, KRB buffer and biofilms were aspirated and replaced by treatment or control samples in KRB respectively and then incubated for 24 hours.

The following control groups were included, infected untreated Calu-3 cells, uninfected Calu-3 cells and Calu-3 cells treated with Triton-X 100 (dead control). Treatment groups that corresponded to infected Calu-3 cells were treated with either free 3C, 3C + HIPS-1635, 3C NP-Lip, 3C NP-Lip + HIPS-1635 and 3C NP-H1635-Lip. Concentration of 3C used was 5 μg/ml.

After incubation, the antibacterial effectiveness of formulations was determined using the colony forming unit assay (CFU) relative to the controls [26]. The toxicity towards Calu-3 cells was determined by the lactate dehydrogenase (LDH) assay [27].

#### Statistics

Statistical analysis was conducted using GraphPad Prism 9 software. Comparisons between two groups were done using Student’s *t* test, while comparisons between several groups were done using analysis of variance (ANOVA) test, where ns indicates non significance, *P < 0.05, **P < 0.01, ***P < 0,001 and ****P < 0.0001.

## Results and discussion

### Formulation and characterization of nanoparticles, liposomes and nanoparticles in liposomes

**Fig. 1:**
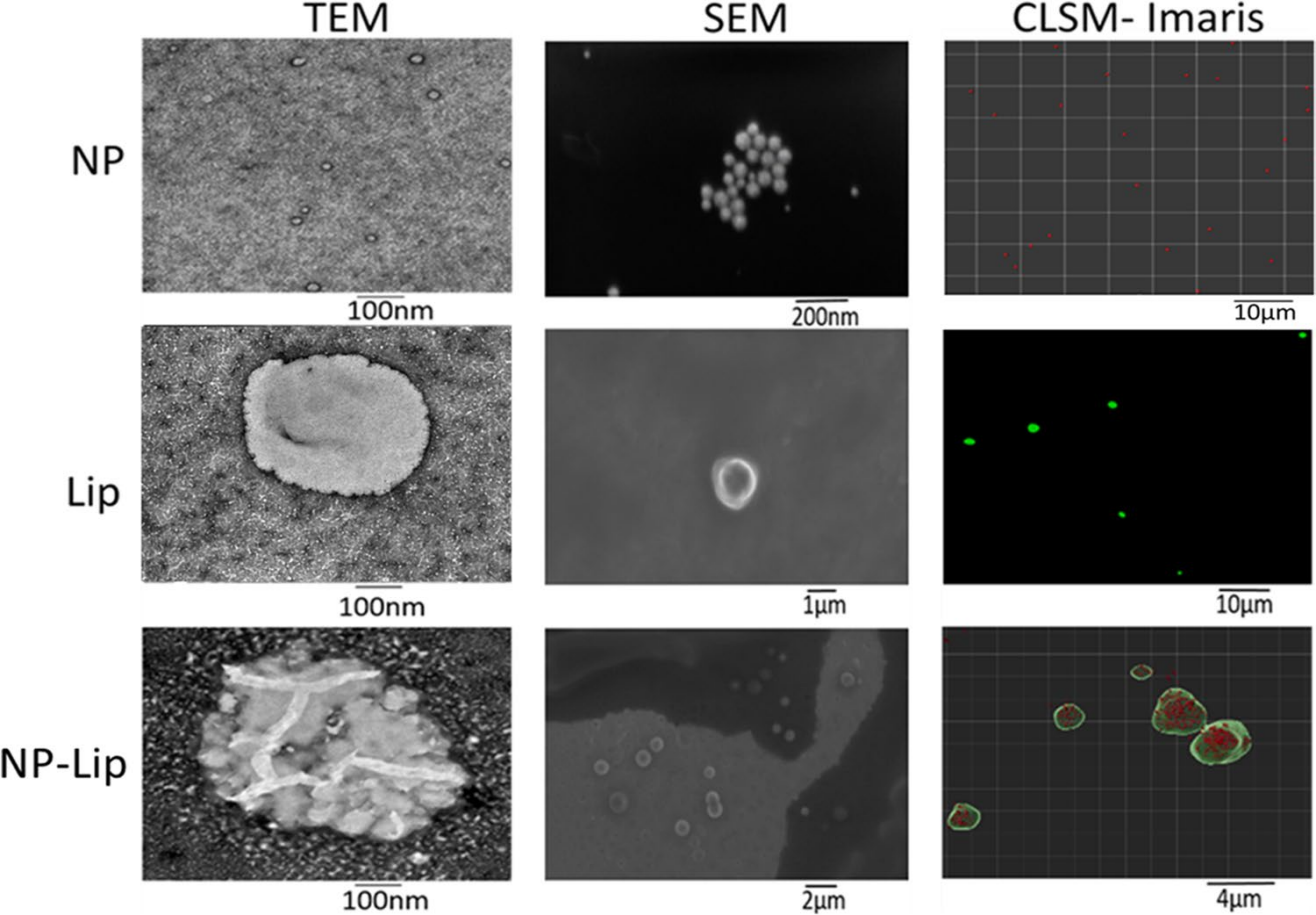
TEM, SEM and CLSM images of NPs, Lip and NP-Lip. For TEM images, NP, Lip and NP-Lip images were captured at magnification of 60,000. For SEM images, NP images were captured at a magnification of 85,580, Lip images were captured at a magnification of 10,000 and NP-Lip images were captured at magnification of 5000. Images of particles with different magnifications are available in Fig. A in the supplementary material

NPs of small size were produced and appeared spherical under TEM, SEM and under CLSM, (Fig. 1). NPs had a negative ZP which was endowed by PLGA’s terminal carboxyl groups [28], and given the non-ionic nature of the surfactant used [29]. Similarly spherical Lip, exhibited negative ZP, due to the fact that the majority of phospholipids in soybean lecithin are also negatively charged [30]. Upon incorporation of NP into Lip, NP-Lip increase in HD was observed. This increase in size was also observed under SEM and TEM. Under CLSM, Lip were excited with the green laser due to their Top-Fluor cholesterol content and NPs with the red laser due to their rhodamine B content. Indeed, CLSM images show NPs appearing red, incorporated within a green bilayer. Images were reconstructed using Imaris software (please see supplementary materials for details). Table 1 shows the HD and ZP of particles.

**Table 1:** Hydrodynamic diameter and zeta potential of different formulations

Particle Type	Mean HD (nm) ± SD	Mean ZP (mV) ± SD
NPs	91.3 ± 3.1	−21.0 ± 0.6
Lip	1019.0 ± 8.5*	−59.0 ± 1.2
NP-Lip	1378.7 ± 106.9*	−40.8 ± 0.6

*P value = 0.0044

Statistical analysis was preformed using Graph Pad software using Student’s *t* test. A value of *p* < 0.05 was considered as indicative of statistically significant difference

The incorporation of NP in Lip was initially confirmed upon the increase in HD of NP-Lip, this was further elucidated upon viewing the CLSM images. In addition to the qualitative confirmation of NP incorporation in Lip, it was necessary to compute I% which corresponded to 71.02% ± 0.080. The relatively high incorporation is a result of Lip formation during the suspension of lipids in the NP suspension in the vortex agitation method.

### In vitro evaluation of NP and NP-Lip cell cytotoxicity and cell uptake

**Fig. 2:**
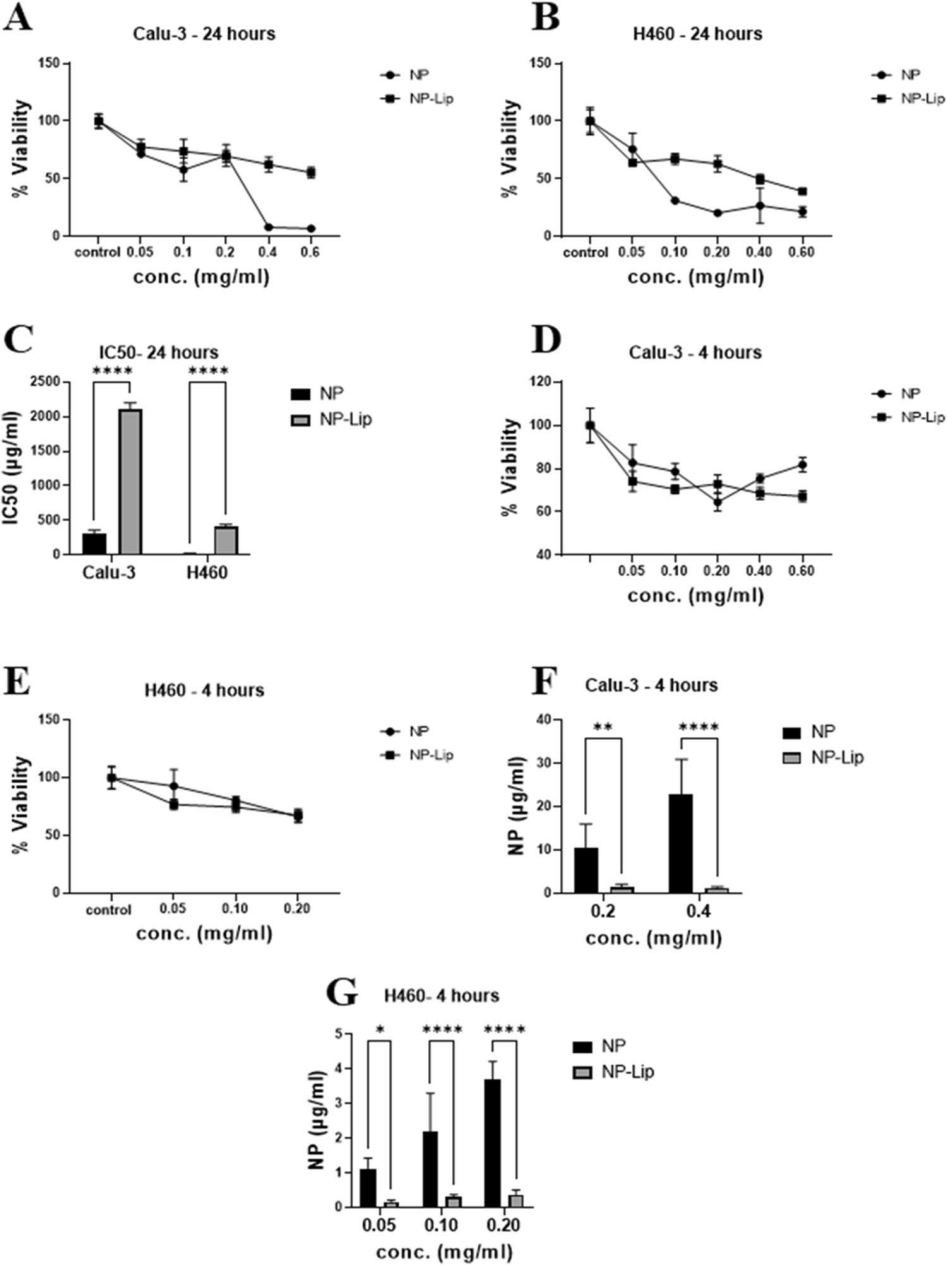
In-vitro assessment of NP and NP-Lip. Cell viability determination following treatment of (**A**) Calu-3 and (**B**) H460 cells with NP and NP-Lip for 24 hours. (**C**) IC50 of NP and NP-Lip towards Calu-3 and H460 cells after 24 hours. Cell viability determination following treatment of (**D**) Calu-3 and (**E**) H460 cells with NP and NP-Lip for 4 hours. Extent of particle uptake after 4 hours by (**F**) Calu-3 cells and (**G**) H460 cells. Statistical analysis was preformed using Graph Pad software using two-way ANOVA where ****P < 0.0001 when comparing IC50 values between NP and NP-Lip as shown in **C**. In **F** and **G** NPs and NP-Lip were compared to each other at each tested concentration using t-tests where *P < 0.05, **P < 0.01, and ****P < 0.0001 (*n* = 6)

Two cell lines were selected; Calu-3 and H460 cells both of which were non-small cell lung cancer cells [31]. Calu-3 cells are able to form monolayers, in addition to their ability to secrete mucus and hence could mimic lung epithelial barrier models [32]. Prior to assessing the extent of uptake, the cytotoxicity of unloaded NPs and NP-Lip was first assessed using concentrations of 0.05, 0.1, 0.2, 0.4 and 0.6 mg/ml in terms of PLGA content (Table 2). Figure 2(A) and (B) shows % viability of Calu-3 and H460 cells respectively following incubation with NPs and NP-Lip for 24 hours. It was evident that upon treatment with increasing concentrations of NPs, after 24 hours, greater loss of viability of Calu-3 and H460 was observed relative to NP-Lip. Upon treatment with increasing concentrations of NP-Lip, no decline in viability was observed (relative to untreated controls) except at higher concentrations (0.4 and 0.6 mg/ml), concluding that NP-Lip had a higher safety profile than NPs on both cell lines. Figure 2(C) shows the IC50 values for NP and NP-Lip in both cell lines corresponding to 309.5 ± 48.6 and 2110.9 ± 90.6 μg/ml respectively towards Calu-3 cells and 22.3 ± 3.2 and 406.5 ± 36.6 μg/ml respectively on H460 cells further confirming the increased safety of NP-Lip. While the toxicity of unloaded PLGA NPs was not to be expected given the relative safety reported numerously in literature [33–35], a possible explanation could be attributed to the excess Tween 80 that was not entirely removed by centrifugation filter units used in NP concentration, which was previously proven toxic on HEK293 cells [11]. The incorporation of NPs in Lip could lower toxicity towards mammalian cells by two mechanisms (1) reduction of free Tween 80 content, since NP-Lip are concentrated by centrifugation not filtration units and hence a significant portion of Tween 80 will not end up in the pellet and (2) larger Lip can lower NPs intracellular concentrations in mammalian cells while also fusing with the bacterial outer membrane, improving antimicrobial activity [12]. To prove the latter, uptake studies were conducted. However, uptake studies had to be conducted using conditions that did not result in significant cell death to avoid clouding of results. For such reasons the cell viability evaluation was repeated after 4 hours incubation. Figure 2(D) and (E) show minimal losses in viability upon incubation with increasing concentrations of NPs and NP-Lip for 4 hours. Accordingly, uptake studies were conducted with rhodamine loaded NPs and NP-Lip following 4 hours incubation and with concentrations that did not cause significant toxicity. Therefore, uptake was conducted with 0.2 and 0.4 mg/ml in terms of PLGA content for Calu-3 cells and H460 cells were treated with 0.05, 0.1 and 0.2 mg/ml in terms of PLGA content. As seen in Fig. 2(F) and (G), as the concentration of NPs and NP-Lip applied increases, the higher the uptake observed. However, it was evident that NP-Lip had significantly lower uptake in cells in comparison to NPs alone in both Calu-3 and H460 cells, hence contributing to the safety of NP-Lip. The rationale behind this observation is that smaller particles have higher internalization causing accumulation of particles and higher toxicity in cells in comparison to larger sized particles which show limited internalization [14]. It is also worth mentioning, that it was proven in literature that PLGA NPs have had an impact on lung cells leading an increase in inflammatory response [36, 37]. Therefore, in future work we aim to evaluate inflammatory markers in both cell lines upon treatment with formulations.

**Table 2:** IC50 values of NPs and NP-Lip towards Calu-3 and H460 cell lines

IC50 (μg/ml)	Calu- 3	H460
NP	309.5 ± 48.6	22.3 ± 3.2
NP-Lip	2110.9 ± 90.6	406.5 ± 36.6

### Evaluation of the suitability of NP-Lip for pulmonary drug delivery

The evaluation of appropriateness of this delivery system for pulmonary delivery was crucial. Within this context three aspects were evaluated (1) the lung deposition pattern of NP and NP-Lip following nebulization, since the formulation should ultimately deliver the drugs deep into the lung (2) NP and NP-Lip behavior in mucus. (3) Since the formulation should reside in the lung and hopefully show minimal absorption into the systemic circulation the permeation of delivery systems across a pulmonary epithelial barrier was evaluated.

**Fig. 3:**
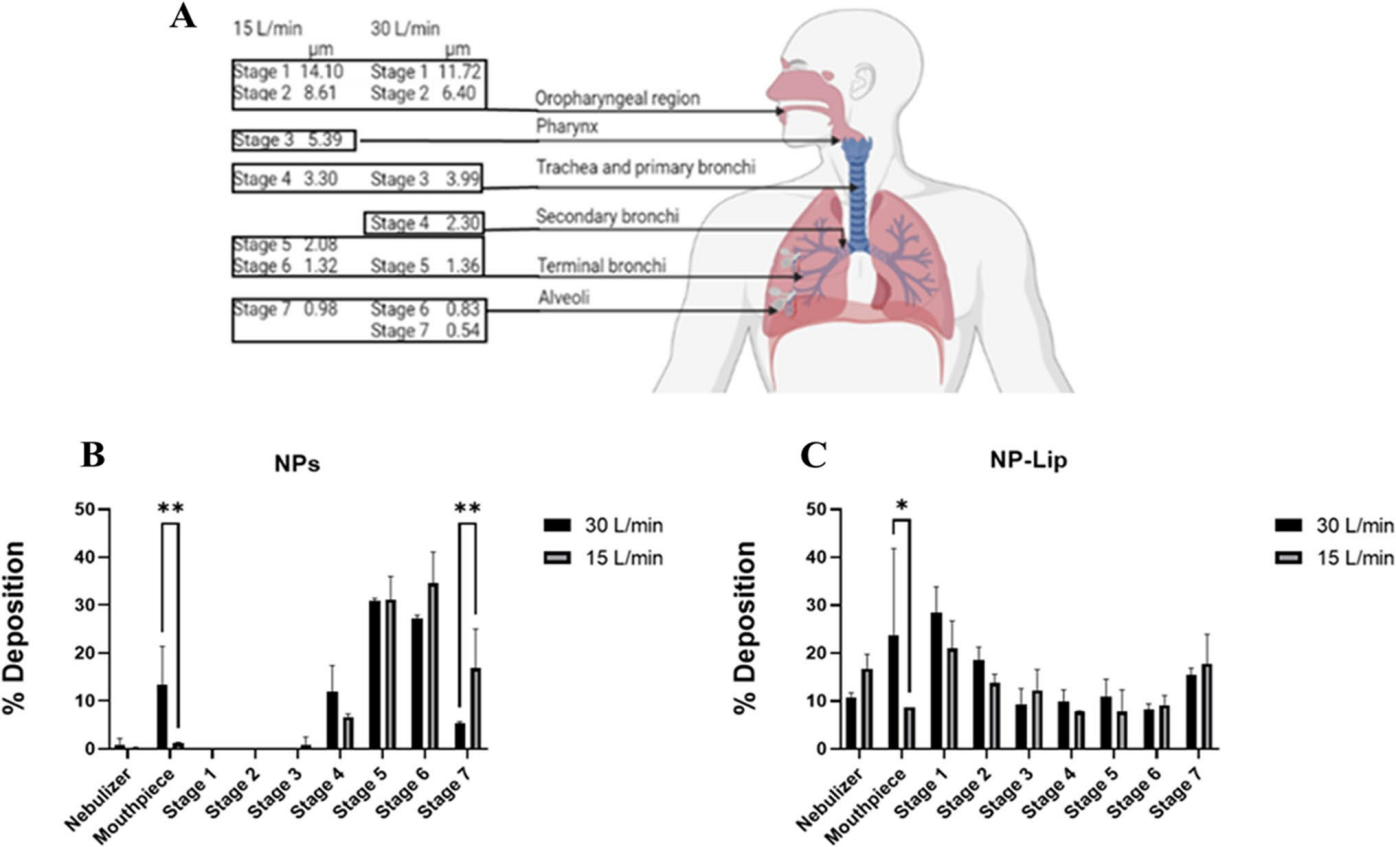
(**A**) Schematic diagram describing the relation of particle aerodynamic diameter and its deposition pattern in the NGI and the correspondent area in the lung. Image was created by Biorender.com. (**B**) % deposition of nebulized NPs at 30 L/min and 15 L/min. (**C**) % deposition of nebulized NP-Lip at 30 L/min and 15 L/min. Statistical analysis was preformed using Graph Pad software using t-test where in (**B**) % deposition was compared at 30 L/min and 15 L/min at the mouthpiece and stage 7. In (**C**) deposition were compared at 30 L/min and 15 L/min at the mouthpiece (*n* = 3)

NP and NP-Lip were nebulized at two different flow rates 30 L/min and 15 L/min as recommended by Copley Scientific for inhalers and nebulizers respectively [38]. Figure 3(B) and (C) show that at 30 L/min the flow rate was rather high, resulting in the deposition of a large amount of NPs (13.3%) and NP-Lip (23.6%) in the mouthpiece. A reduction of the flow rate to 15 L/min has reduced deposition in the mouthpiece enabling a larger portion of the formulation to make it into the respiratory system. The MMAD of NPs at 15 L/min and 30 L/min was 1.48 μm and 1.17 μm respectively, meanwhile the MMAD of NP-Lips at 15 L/min and 30 L/min was 4.41 μm and 3.30 μm respectively. Figure 3(B) shows that NPs were capable of depositing deep into the lung. In fact, NPs % deposition in stages 3–7 collectively at flow rate 30 L/min was 72.122% ±0.77 and 77.492% ± 4.47 at 15 L/min. For NP-Lip % deposition in stages 3–7 at flow rate 30 L/min was 53.765% ±0.13 and 54.985% ± 4.52 at 15 L/min. These numbers suggest that a reduced flow rate or a dilution of the nebulized NP-Lip suspension could have reduced NP-Lip deposition in stages 1–2. While it seems that from a lung deposition perspective NPs outperform NP-Lip, an important point to consider is that NPs (due to their smaller aerodynamic diameter) have greater tendency to reach the alveoli and coupled with their small size would translate into greater ability in permeating into the systemic circulation [39]. Upon nebulization particles maintain their same size with the folds change of particle size equal to 1.02.

**Fig. 4:**
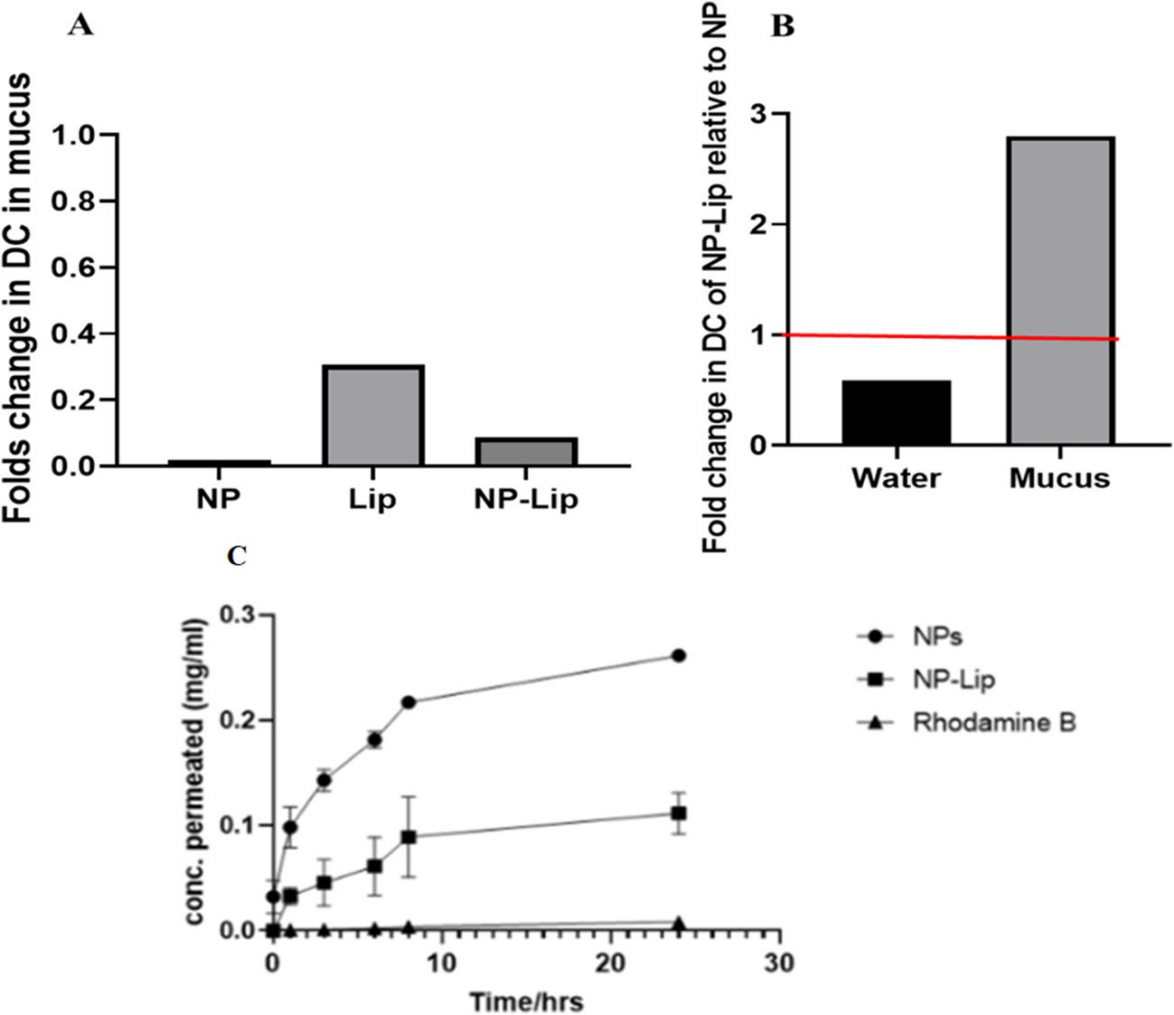
(**A**) Folds change in diffusion coefficient of NPs, Lip and NP-Lip in mucus relative to water. (**B**) Folds change in diffusion coefficient of NP-Lip relative to NPs in water and mucus surrogate. (**C**) Permeation of NP, NP-Lip and Rhodamine B

It was necessary to adopt a mucus surrogate that allowed for the size-filtering features, and a dense structure to best mimic mucus in cystic fibrosis and therefore artificial mucus was used. The diffusion coefficient of particles was calculated with the use of multiple particle tracking analysis after the suspension in water and in mucus and according the calculations in [40]. Figure 4(A) presents the results as folds change in diffusion coefficient of NP, Lip and NP-Lip achieved through incorporation of mucus. Since all values are lower than one, this indicated that NPs, Lip and NP-Lip show a reduction in diffusion in mucus relative to water. The latter was rather expected given the increased viscosity and size filtering properties of mucus [41]. In Fig. 4(B), the diffusion coefficient of NP-Lip was divided by that of NPs in different media. In water, the value is smaller than one, indicating slower movement of NP-Lip relative to NPs, which is to be expected given the larger particle size of NP-Lip relative to NP [40]. In mucus, however, interestingly, when NPs were incorporated in Lip, NP-Lip diffusion increased in mucus (Fig. 4(B)). A possible explanation is that PLGA is mucoadhesive [42] and hence its encapsulation in Lip shields the PLGA surface allowing for increased mobility of particles in mucus.

In order to limit systemic toxicity while treating pulmonary infections, therapeutic molecules should be retained in lungs and exhibit minimum permeation [43]. Upon treatment of cells with NPs, higher permeation was observed in comparison to treatment with the same concentration of NP-Lip as shown in Fig. 4(C). This observation was consistent with previous findings indicating that smaller sized particles generally have higher permeation kinetics than larger sized particles [39]. Rhodamine B dye was also applied to cells and had the least permeation, confirming that any observed transport was indeed due to particles and not just leaking of rhodamine B dye. Accordingly, results indicate that NP-Lip showed a higher safety profile and could potentially show less systemic toxicity relative to NPs.

### Evaluation of the ability of NP-Lip to increase antibiotic effectiveness and selectivity in *P. aeruginosa* biofilms

**Fig. 5:**
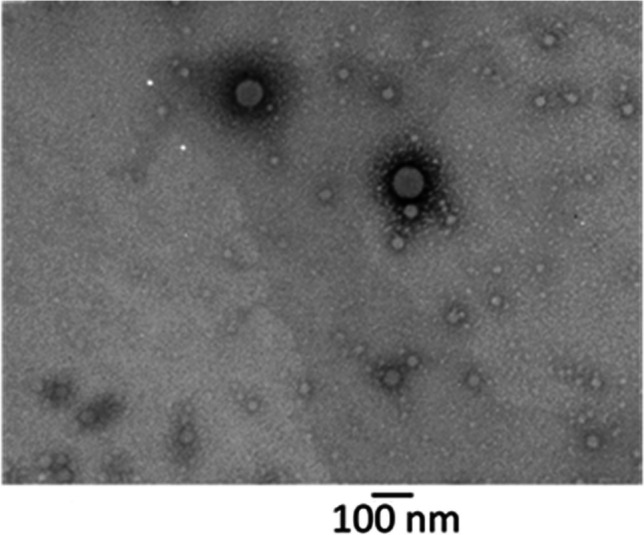
TEM image of 3C NP

3C and Tbr were encapsulated in NPs, while HIPS-1635 was encapsulated in Lip. 3C had a relatively low EE% of 25%, equivalent to 5 μg/ml. Although this EE% was not very high, the concentration of the encapsulated 3C matched the reported MIC50 for the *P. aeruginosa* in this work, making it acceptable. In contrast, Tbr also had a low EE% of 23.8%, equivalent to 9.5 μg/ml, which was expected due to its hydrophilic nature [44]. Using a hydrophilic polymer for encapsulation would have improved Tbr’s EE%, but our primary focus in this work was on 3C, justifying the use of PLGA. HIPS-1635 demonstrated a higher EE% of 52%, equivalent to 104.5 μg/ml in Lip. This higher efficiency was expected as HIPS-1635 is hydrophobic and would integrate into the phospholipid bilayer of the Lip [45]. After the loading of drugs into delivery systems, NP size corresponded to 122.6 ± 11.4 nm, while NP-Lip was 1391.3 ± 113.4 nm. Figure 5 shows the morphology of 3C NP, confirming no change in NP morphology after drug loading and therefore it can be assumed that drug loaded NP-Lip morphology would also not change.

**Fig. 6:**
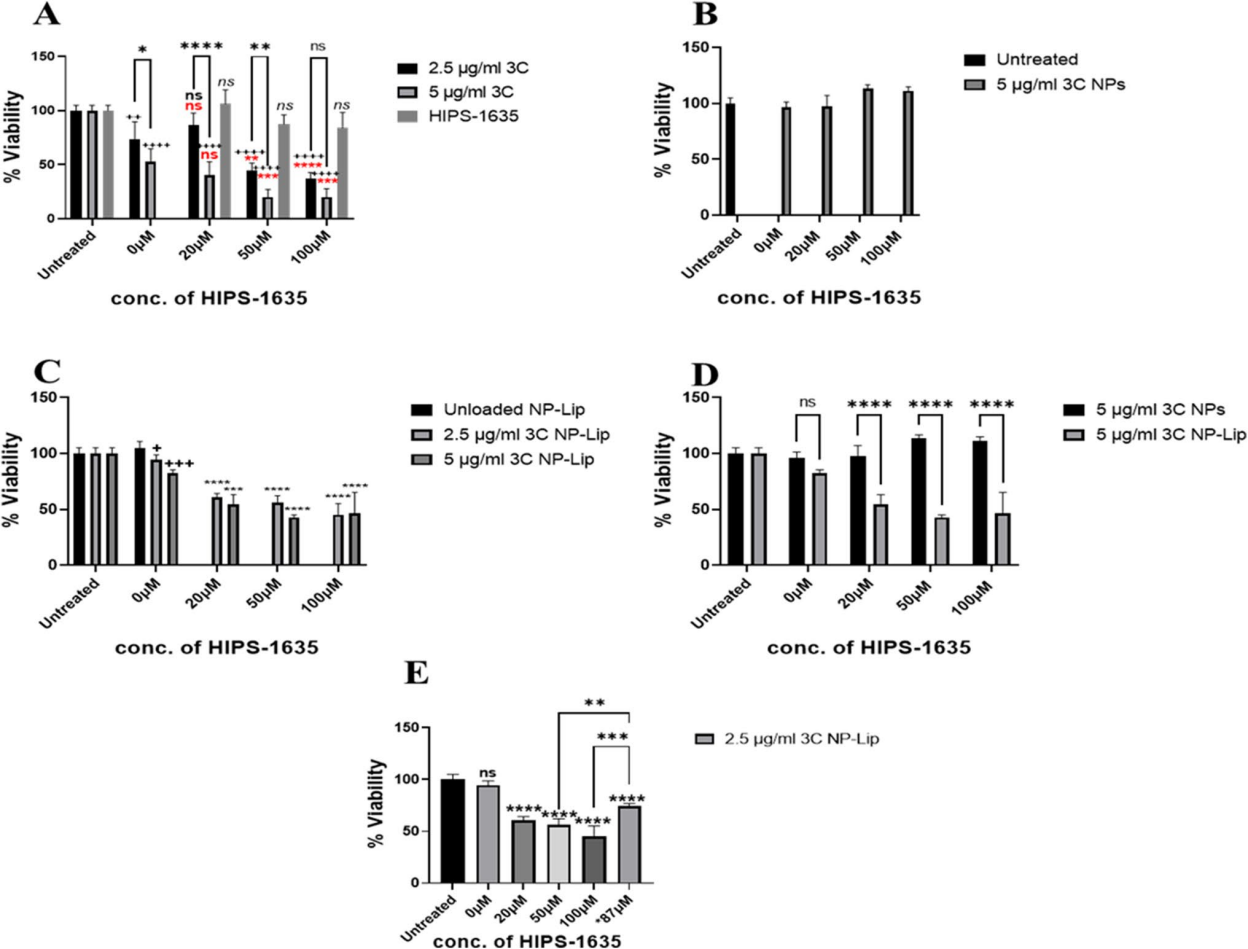
Viability of *P. aeruginosa* biofilms upon treatment with (**A**) free unencapsulated 3C alone and in combination with HIPS-1635, (**B**) 3C loaded NPs alone and in combination with HIPS-1635, (**C**) unloaded and loaded 3C NP-Lip alone and in combination with HIPS-1635, (**D**) comparison between 3C NP and 3C NP-Lip, (**E**) comparison between 3C NP-Lip with HIPS-1635 in its unencapsulated and encapsulated form (indicated as *87 μM). Statistical analysis was conducted by Graph Pad using two-way ANOVA. In (**A**) the statistical comparisons indicated by brackets indicate comparison between biofilms treated with 2.5 μg/ml 3C and 5 μg/ml 3C groups. Statistical comparisons not indicated by brackets indicated in red show the significance between 3C alone and 3C with different concentrations of HIPS-1635. The + and black statistics not indicated by brackets indicate significance of treated groups relative to untreated control. In (**C**) The + indicates significance between unloaded NP-Lip and NP-Lip loaded with the different 3C concentrations, while the rest of the statistics are conducted to show significance between 3C NP-Lip in comparison to 3C NP-Lip with HIPS-1635. In (**E**) all statistical comparisons are compared to untreated controls, unless indicated by brackets (*n* = 5)

To assess the effectiveness in a quantitiative manner, the presto blue assays have shown the loss of viability of *P. aeruginosa* biofilms upon treating with free 3C with its MIC50 reported in this work which is equivalent to 5 μg/ml, and upon dual therapy with 3C and HIPS-1635 in their unencapsulated form. The latter indicates the complementarity of both compounds, due to the potentiation of toxicity as shown in Fig. 6(A). In Fig. 6(B), upon treatment with 3C loaded NPs, loss of biofilm viability did not occur, however interestingly (Fig. 6(C)), upon loading with increasing concentrations of 3C in NP-Lip, a significant increase in toxicity was seen in comparison to unloaded NP-Lip. This effect was further potentiated with the use of HIPS-1635 in its unencapsulated form along with 3C NP-Lip (3C NP-Lip + HIPS 1635). This would be justified by the fusion of Lip bilayers with the outer membrane allowing the entry and diffusion of 3C NP through biofilms [46]. In fact, the superior bactericidal effect of NP-Lip could therefore be attributed to a better ability to deliver 3C into bacterial cells. To compare between NPs and NP-Lip as shown in Fig. 6(D), 3C NP-Lip has shown significantly higher toxicity towards *P. aeruginosa* biofilms along with the increasing concentrations of HIPS-1635 in comparison to 3C NPs. It is worth mentioning, that in the previous charts (Fig. 6(A)-(D)), the HIPS-1635 was unencapsulated, and it was merely used in its free form. When the experiment was repeated with HIPS compound inside the 3C NP-Lip (3C NP-H1635-Lip), significant reduction of bacterial viability relative to untreated control was observed, however its toxic effect was not as prominent as that with the 3C NP-Lip + HIPS 1635. This could be justified by the direct ability of unencapsulated HIPS compound to act, while the encapsulated form would still require its release from the Lip, and not due to the reduced effectiveness of this delivery system [47]. Its encapsulation would however guarantee that following nebulization, HIPS-1635 would allocate in the same location as 3C given their loading into the same carrier system and hence bring out a complementary effect [47].

**Fig. 7:**
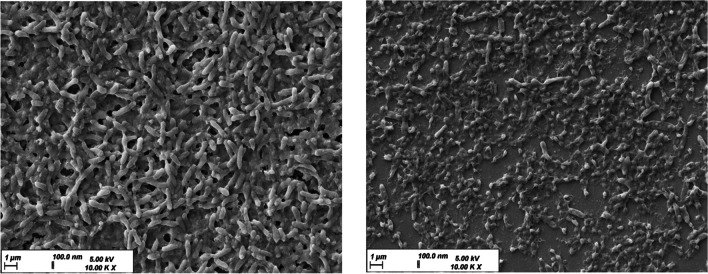
SEM images of biofilm (**A**) untreated, (**B**) treated with 3C NP- H1635- Lip

To further confirm the results of presto blue assay, a more qualitative mean of analysis was utilized in which biofilm images were captured using SEM before and after treatment with the most promising treatment group 3C NP- H1635- Lip (Fig. 7). Images show that upon treatment, loss of biofilm integrity occurred along with loss of bacterial viability. This could confirm that the NP-Lip delivery system shows prominent antibacterial effects, but whether this is a selective approach is to be determined according to the studies conducted on lung cells.

**Fig. 8:**
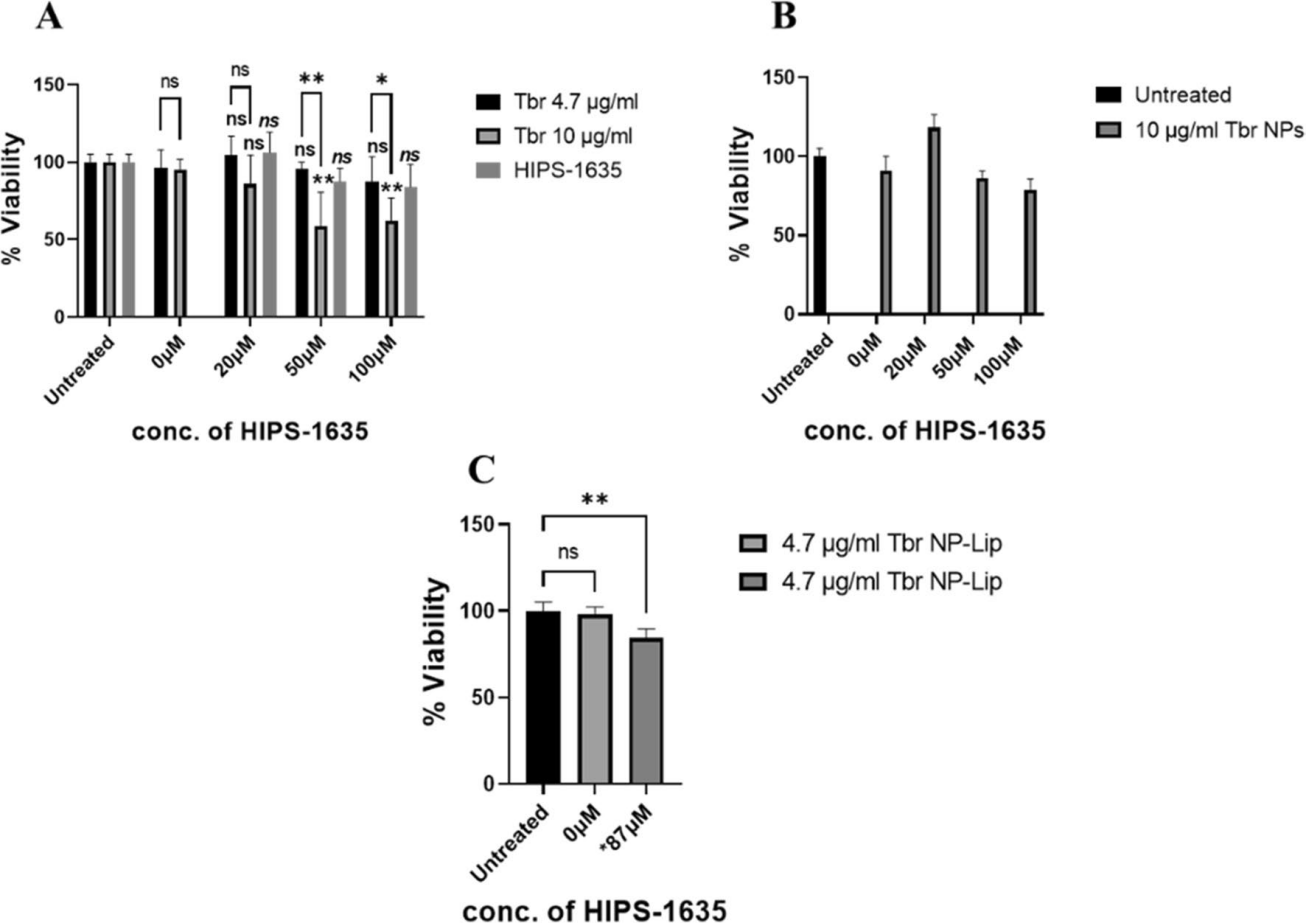
Viability of *P. aeruginosa* biofilms upon treatment with (**A**) free Tbr alone and in combination with HIPS-1635, (**B**) Tbr loaded NPs with co-treatment with HIPS-1635, (**C**) Tbr NP-Lip with HIPS-1635 in its encapsulated form (Tbr-NP-H1635-Lip). Statistical analysis was conducted by Graph Pad using two-way ANOVA. In (**A**) statistics in brackets indicate significance between 4.7 μg/ml Tbr groups with the 10 μg/ml Tbr groups, while the rest of statistics indicate the significance between Tbr alone and Tbr with different concentrations of HIPS-1635. The italicized *ns* on HIPS groups indicate non-significance relative to untreated control (*n* = 5)

The same experiments were then repeated, but this time with the already established antibiotic Tbr. According to literature the MIC90 of Tbr against *P. aeruginosa* was 4 μg/ml and hence why the tested concentrations were chosen [48]. As shown in Fig. 8(A), the antibiotic Tbr with the reported MIC90 or double that concentration alone, did not show any toxic effect on *P. aeruginosa* biofilms. The latter has been previously reported, due it’s overall positive charge, Tbr is sequestered to the biofilm periphery. In contrast neutral antibiotics such as ciprofloxacin readily penetrated similar biofilms [49]. Similar to ciprofloxacin, 3C is also a neutral antibiotic, it’s superior bactericidal effects could in part be attributed to its increased ability to penetrate biofilms relative to Tbr therefore contributing to the more prominent toxic effect. When Tbr was applied with a higher concentration (10 μg/ml) in combination with the higher concentrations of HIPS-1635 (50 and 100 μM), significant loss of viability was evident. This confirmed the complementarity of Tbr and HIPS-1635 at higher concentrations that was previously proven [17]. Similar to results observed with 3C, upon treatment with increasing concentrations of Tbr NPs, no toxicity was evident even when combined with increasing concentrations of HIPS-1635 (Fig. 8(B)). In Fig. 8(C), a significant loss of viability upon encapsulation of Tbr NP in Lip containing the HIPS-1635 (Tbr NP- H1635-Lip) was evident, in comparison to the untreated control. Although reduction in bacterial viability was more modest than that seen with 3C, this once more confirms the superiority of NP-Lip to NPs.

### Assessing the cytotoxicity of drug loaded NPs and NP-Lip towards Calu-3 cells

**Fig. 9:**
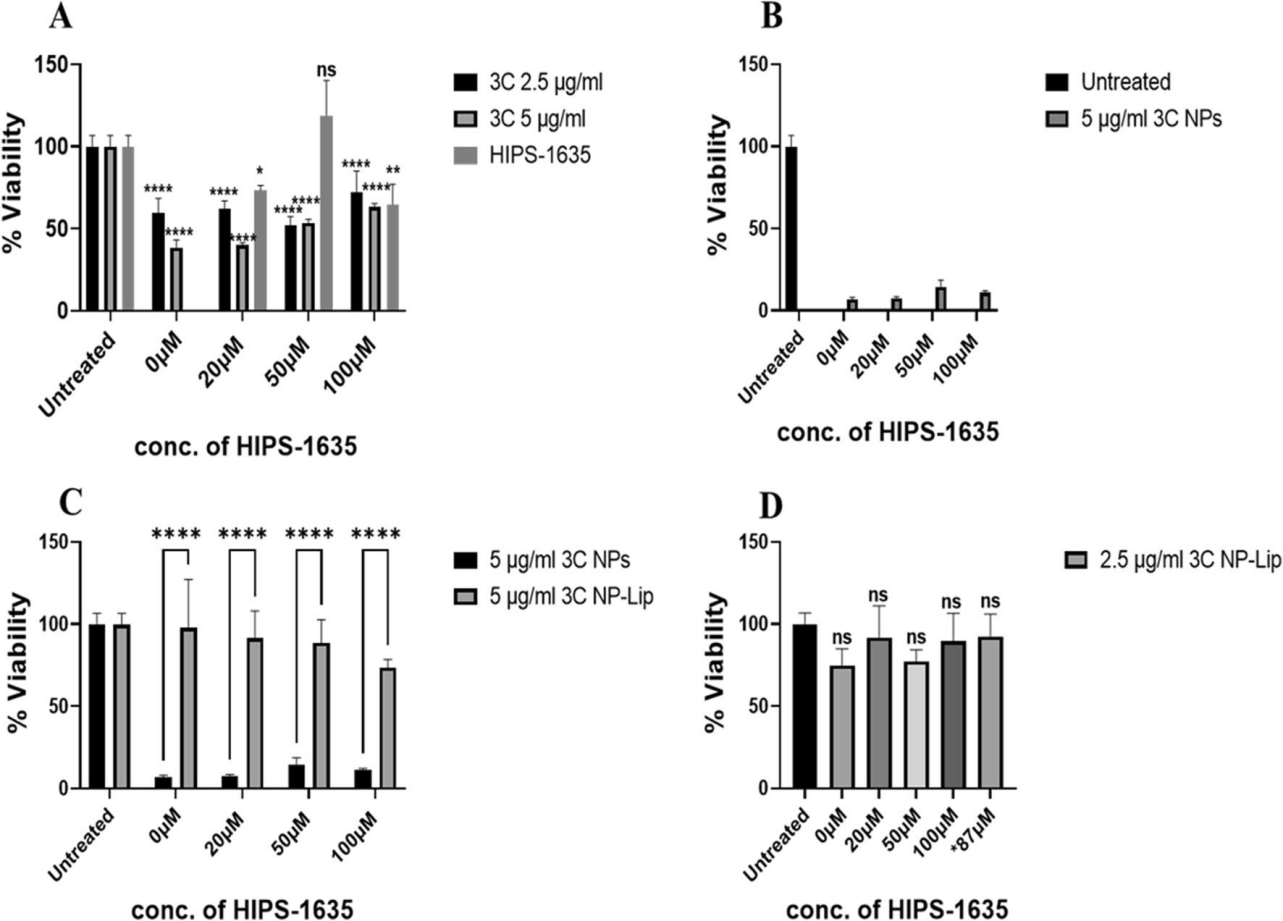
Viability of Calu-3 upon treatment with (**A**) unformulated 3C alone and in combination with HIPS-1635, (**B**) loaded 3C NPs alone and in combination with HIPS-1635, (**C**) comparison between 3C NP and 3C NP-Lip, (**D**) comparison between 3C NP-Lip with HIPS-1635 in its unencapsulated and encapsulated form (indicated as *87 μM). Statistical analysis was conducted by Graph Pad using two-way ANOVA. In (**A**) and (**D**) all groups were compared to untreated control while In (**C**) all 3C NP groups were compared to 3C NP-Lip (*n* = 5)

After the determination of the antibacterial effectiveness of NPs and NP-Lip, the cytotoxicity of the antibiotic loaded formulations towards Calu-3 cells was assessed in order to determine whether an increase in antibiotic selectivity is achieved. Figure 9(A) shows that a significant reduction in viability of Calu-3 cells was observed when treated with free 3C and free HIPS-1635 whether separately or in combination. This indicates the high toxicity of free drugs and the necessity behind their encapsulation in carriers to decrease their toxic effect. 3C loaded NPs have also exhibited high cytotoxic effect as shown in Fig. 9(B), due to the increased delivery and internalization of 3C into the Calu-3 cells [50]. Meanwhile in Fig. 9(C), 3C NP-Lip showed significantly lower reduction in cell viability in comparison to 3C NPs either alone or with HIPS-1635. This confirmed the high safety profile of NP-Lip which is justified by the decreased internalization inside the cells due to the large size of NP-Lip, therefore limiting the toxicity [14]. It is also worth noting, that whether the HIPS compound was encapsulated in the NP-Lip delivery system (3C NP-H1635- Lip) or in its unencapsulated form (3C NP-Lip + HIPS-1635), did not result in significant cytotoxic effect relative to untreated control as evident in Fig. 9(D). This concludes the high selectivity of 3C NP-H1635-Lip since it shows no significant cytotoxic effect towards the Calu-3 cells, however shows high antibacterial effectiveness towards *P. aeruginosa* biofilms.

**Fig. 10:**
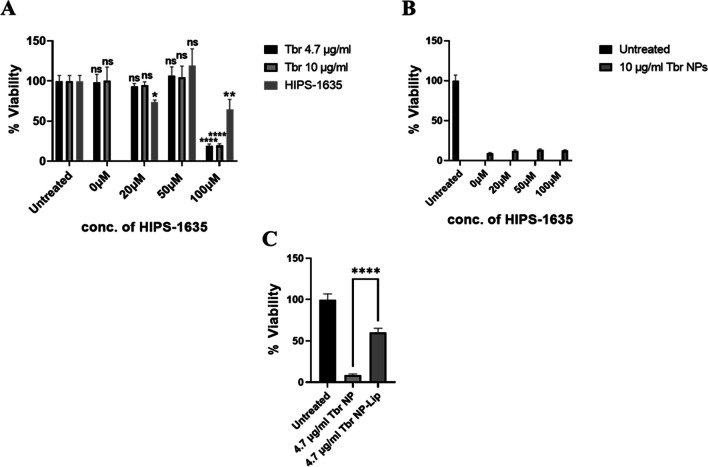
Viability of Calu-3 upon treatment with (**A**) unformulated Tbr alone and in combination with HIPS-1635, (**B**) loaded Tbr NPs alone and in combination with HIPS-1635, (**C**) comparison between Tbr NP and Tbr NP-Lip. Statistical analysis was conducted by Graph Pad using two-way ANOVA. In (**A**) all groups were compared to the untreated control, while in (**C**) Tbr NPs were compared to Tbr NP-Lip (*n* = 5)

Experiments were then repeated with the Tbr formulations to assess whether similar results would be obtained. Figure 10(A) shows that with the highest concentrations of HIPS-1635 very significant cytotoxicity is observed when cells were treated with Tbr at both 4.7 and 10 μg/ml. This toxicity is not observed when cells were treated with Tbr only, or Tbr in combination with lower concentrations of HIPS-1635. Tbr loaded NPs have also exhibited high cytotoxic effects as shown in Fig. 10(B), due to the increased uptake of Tbr into Calu-3 cells as a result of its encapsulation in small NPs [50]. In Fig. 10(C), a comparison in the viability of Calu-3 cells was assessed upon treatment with Tbr NPs and Tbr NP-Lip. Results highlighted that while Tbr NP-Lip still showed loss of viability in comparison to the control untreated groups, however this cytotoxic effect was still significantly lower to Tbr NPs. This further confirms the benefit of NP-Lip formulation for the safety of cells and the toxicity towards bacterial biofilms therefore, offering selectivity.

### Co-culturing Calu-3 cells with *P. aeruginosa* biofilm

**Fig. 11:**
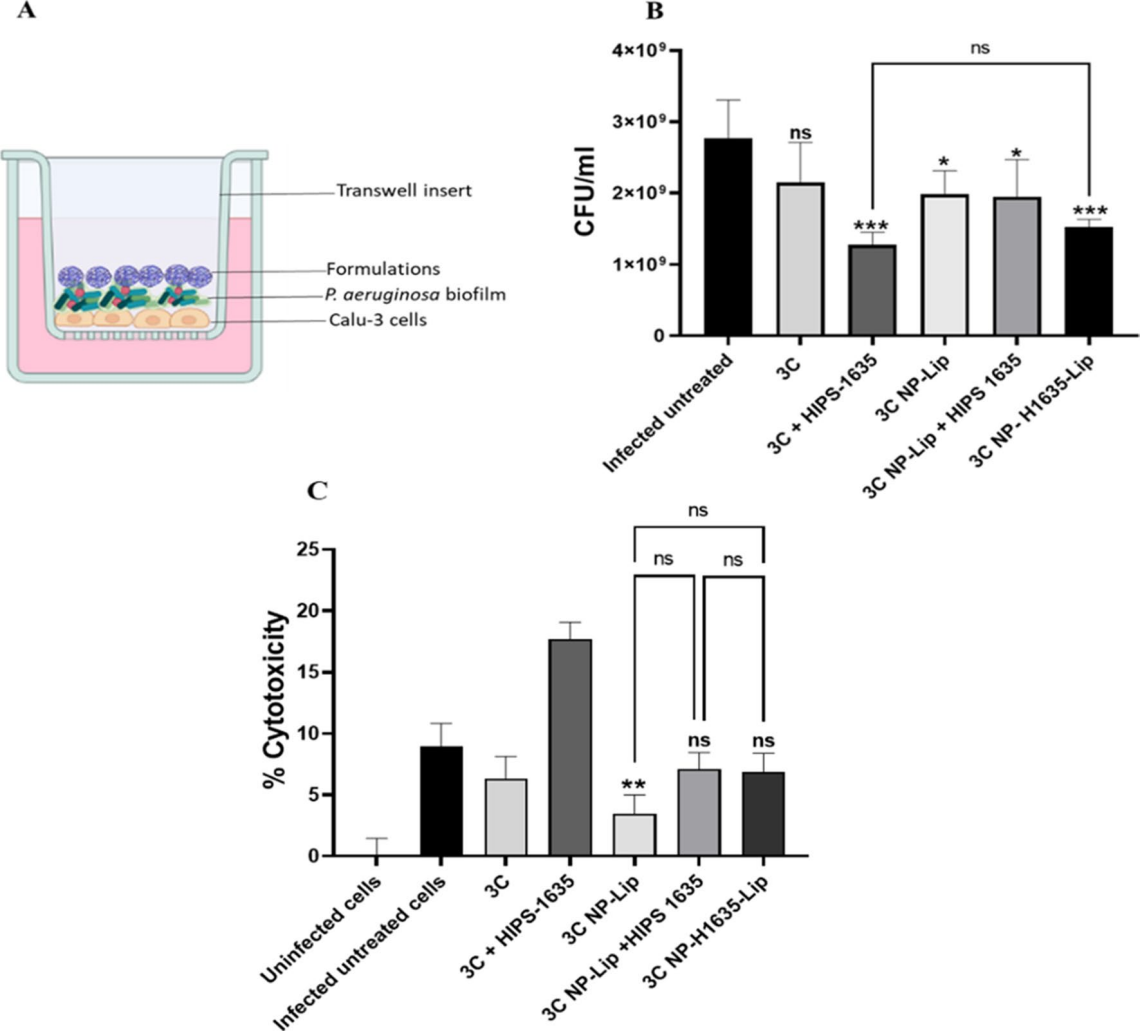
(**A**) Schematic representation of co-culture set-up, (**B**) Viability of *P. aeruginosa* and (**C**) % Cytotoxicity on Calu-3 cells upon treatment with different 3C formulations in co-culture model. Statistical analysis was conducted by Graph Pad using one-way ANOVA. In (**B**) all treatment groups were compared to the infected untreated groups and in (**C**) 3C NP-Lip, 3C NP-Lip + HIPS and 3C NP-H1635-Lip treatment groups were compared to infected untreated Calu-3 cells. Statistics shown in brackets indicate significance of groups to each other (*n* = 3)

Due its superior bactericidal effects, 3C loaded systems were subjected to further testing in co-culture experiments. In co-culture models the natural communication between the bacterial and mammalian cells is taken into account. These systems therefore allow a better understanding of human- in vivo interactions in comparison to the monocultured systems [51]. In comparison to untreated biofilms grown in the presence of Calu-3 cells, when biofilms were treated with 3C alone in its unformulated state, a significant toxic effect on *P. aeruginosa* was not observed which was contradicting to the previous results observed in monocultures (Fig. 6(A)). This could be explained by the competition for uptake of 3C between bacterial and lung cell when both are cultured together [52]. However, a significant reduction in bacterial viability was observed upon combining 3C with the HIPS compound. 3C NP-Lip and 3C NP-Lip with HIPS-1635 in its unencapsulated form showed moderate reduction in CFU/ml, while 3C NP-Lip encapsulating HIPS-1635 (3C NP-H1635-Lip) showed the more prominent effect on bacterial toxicity. Since free HIPS-1635 has indeed resulted in cell viability losses in Calu-3 cells (Fig. 10(A)), it could be speculated that in co-cultures, some of added HIPS-1635 diffuse into Calu-3 cells, reducing the available HIPS-1635 for biofilm dissipation. It is however worth noting, that the bactericidal effect of free HIPS in combination with free 3C was comparable to that for 3C NP-H1635-Lip. Based on this result only, it becomes questionable whether there is a need for encapsulation of such drugs into NP-Lip. The answer however becomes rather obvious when considering the second half of the experiment; the cytotoxicity towards Calu-3 cells (Fig. 11(C)). The LDH assay was used and results were presented as % cytotoxicity. As shown in Fig. 11(C) all treatment group’s cytotoxicity was compared to that of the infected untreated cells and not the healthy uninfected cells, to account for any cell membrane damage or cytotoxicity imparted by the presence of bacteria with cells [53]. Results have shown that in their free form when 3C + HIPS-1635 were used in treatment, significant cytotoxicity was observed. This was reduced upon encapsulation of the drugs in NP-Lip. While the results displayed in Fig. 11(C) do not show a difference in cytotoxicity between cells treated with 3C NP-Lip, 3C NP-Lip +HIPS-1635 and 3C NP-H1635-Lip, this could be attributed to the use of the LDH assay. Protease production by *P. aeruginosa* results in LDH degradation, making the assay less sensitive [53] Therefore in future work to overcome the sensitivity issue, the modified protocol “intracellular LDH assay” could be utilized in which it depends on the induction of release of LDH from the cells that are still viable. Finally, it was then concluded that drug loaded NP-Lip have exhibited high bactericidal effect in co-cultures and reduced cytotoxicity towards lung cells confirming previous results obtained from monoculture experiments and confirming the high potential of this delivery system in the treatment of biofilm infections in the lungs.

## Conclusion

The NP-Lip delivery system has shown promising results in the selective treatment of *P. aeruginosa* biofilms in the lung while sparing the toxic effect towards lung cells. This was due to the ability of this system to encapsulate both antibiotics and quorum sensing inhibitors, and increase their delivery in bacterial cells. The high antibacterial effectiveness is due to the fusion of the liposomes with outer bacterial membranes and the safety was contributing to the larger size of particles, which reduces uptake in lung cells. Furthermore, this system has shown its suitability for pulmonary delivery which was confirmed by its deposition in the target regions in the lungs, its improved diffusion through barriers such as mucus and its reduced permeation out of the lungs, which could results in less systemic toxicity. In future work, the nebulization of NP-Lip in animal models and assessment of deposition pattern will be conducted. The latter would allow a more realistic output and highlight the role of mucus.

## Supplementary information

ESM 1 (DOCX 1784 kb)

## Data Availability

The datasets generated during the current study are available from corresponding author upon reasonable request.
