# Supplementary material for: Nanoparticles in liposomes: a platform for increased antibiotic selectivity in multidrug resistant bacteria in respiratory tract infections
Source: Drug Deliv Transl Res. 2024 Jul 24;15(4):1193–209. doi: 10.1007/s13346-024-01662-2 (PMC11870967; doi:10.1007/s13346-024-01662-2)
Supplement: Supplementary file 1 — (DOCX 1784 kb) [file 13346_2024_1662_MOESM1_ESM.docx]

# Nanoparticles in Liposomes: A Platform for Increased Antibiotic Selectivity in Multidrug Resistant Bacteria in Respiratory Tract Infections

Nathalie E. Fakhoury ^a^ *, Samar Mansour ^a^, Mohammad Abdel-Halim ^b^, [Mostafa M. Hamed](https://onlinelibrary.wiley.com/authored-by/Hamed/Mostafa+M.) ^c^, Martin Empting ^c^, Annette Boese ^c^, Brigitta Loretz ^c^, Claus-Michael Lehr ^c,d^ , Salma N. Tammam ^a^

a) Department of Pharmaceutical Technology, Faculty of Pharmacy & Biotechnology, the German University in Cairo, Egypt

b) Department of Pharmaceutical Chemistry, Faculty of Pharmacy & Biotechnology, the German University in Cairo, Egypt

c) Helmholtz Institute for Pharmaceutical Research Saarland (HIPS), Helmholtz Center for Infection Research, 66123 Saarbrücken, Germany

d) Department of Pharmacy, Saarland University, 66123 Saarbrücken, Germany

***Correspondences to: nathalie.emad@guc.edu.eg**

**Supplementary file 1- Nanoparticles**

**Dimensions**

| **Dimension** | **Logical Size** | **Physical Length** | **Start Position** | **End Position** | **Pixel Size / Voxel Size** |
| --- | --- | --- | --- | --- | --- |
| X | 512 | 144.72 µm | 0 µm | 144.72 µm | 0.283 µm |
| Y | 512 | 144.72 µm | 10.24 µm | 154.96 µm | 0.283 µm |

**Channels**

| **LUT** | **Resolution** | **Min** | **Max** | **STED: DetectorMode / Huygens saturation factor / Wavelength** |
| --- | --- | --- | --- | --- |
| Green 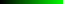 | 8 | 0 | 255 | --- / --- / --- |
| Red 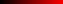 | 8 | 0 | 255 | --- / --- / --- |

**Time Stamps:**

| **Frame (Show All)** | **Relative Time (s)** | **Absolute Time (h:m:s.ms)** | **Date** |
| --- | --- | --- | --- |
| 1 | 0.000 | 10:55:23 AM.449 | 10/4/2022 |
| 2 | 0.000 | 10:55:23 AM.449 | 10/4/2022 |

**Confocal Settings**

| **Name** | **Value** |
| --- | --- |
| Rotator Angle | 0 ° |
| Scan Mode | xyz |
| Scan Direction X | Bidirectional |
| Scan Speed | 700 Hz |
| Version Number | 15 |
| StagePosX | 65,458.54 µm |
| StagePosY | 41,465.1 µm |
| ZPosition | -120.95 µm |
| IsSuperZ | 0 |
| Magnification | 63 |
| ObjectiveName | HC PL APO CS2 63x/1.20 WATER |
|  |  |

| Immersion | WATER |
| --- | --- |
| Numerical Aperture | 1.2 |
| RefractionIndex | 1.33 |
| Zoom | 1.28 |
| Pinhole | 83.5 µm |
| PinholeAiry | 749.52 mAU |
| EmissionWavelength for PinholeAiry Calculation | 580 nm |
| MotCorrPosition | 39.8 °% |
| FrameAverage | 1 |
| LineAverage | 2 |
| FrameAccumulation | 1 |
| Line_Accumulation | 1 |
| IsUserSettingNameSet | 0 |
| IsRoiScanEnable | 0 |

Filter Wheels / Other Motorized Devices

| **Device Name** | **Filter Name/Position** |
| --- | --- |
| Galvo Slider | Galvo X Normal |
| Galvo Resonant Pan | Galvo X Pan Center |
| Target Slider | Target Park |
| X2 Lens Changer | CS2 UV Optics 1 |

Lasers

| **LaserName** | **OutputPower** |
| --- | --- |
| **Diode 405** | **Off** |
| **Argon** | **On, 29.3651 %** |
| **DPSS 561** | **On** |
| **HeNe 633** | **Off** |

Laser Lines

| **Laser Line** | **Intensity** |
| --- | --- |
| ( 405 nm) | Shutter: off, Intensity: 0.0000% |
| ( 458 nm) | Shutter: on, Intensity: 0.0000% |
| ( 476 nm) | Shutter: on, Intensity: 0.0000% |
| ( 488 nm) | Shutter: on, Intensity: 0.0000% |
| ( 496 nm) | Shutter: on, Intensity: 1.0005% |
| ( 514 nm) | Shutter: on, Intensity: 0.0000% |
| ( 561 nm) | Shutter: on, Intensity: 0.0000% |
| ( 633 nm) | Shutter: on, Intensity: 0.0000% |

Detectors

| **Name** | **Channel** | **Type** | **Location** | **Active** | **Gain** | **Offset** | **Gate Start** | **Gate End** | **Gate Ref. Wavelength** |
| --- | --- | --- | --- | --- | --- | --- | --- | --- | --- |
| HyD 1 | Channel 1 | HyD (380nm - 399nm) Standard mode | Internal | Inactive | 100 | -- | -- Time Gating not supported -- | | |
| PMT 2 | Channel 2 | PMT (501nm - 560nm) | Internal | Inactive | 800 | 0 | -- Time Gating not supported -- | | |
| HyD 3 | Channel 3 | HyD (561nm - 566nm) Standard mode | Internal | Inactive | 100 | -- | -- Time Gating not supported -- | | |
| PMT 4 | Channel 4 | PMT (566nm - 586nm) | Internal | Active | 800 | 0 | -- Time Gating not supported -- | | |
| PMT Trans | Transmission Channel | PMT | TLD | Inactive | 0 | 0 | -- Time Gating not supported -- | | |

**Sequential Setting Nr.1**

| HyD 1 | Inactive, Gain: 0, Offset: 0 |
| --- | --- |
| PMT 2 | Active, Gain: 0, Offset: 0 |
| HyD 3 | Inactive, Gain: 0, Offset: 0 |
| PMT 4 | Inactive, Gain: 0, Offset: 0 |
| PMT Trans | Inactive, Gain: 0, Offset: 0 |
| Laser Line ( 405 nm) | Intensity: 0.0000% |
| Laser Line ( 458 nm) | Intensity: 0.0000% |
| Laser Line ( 476 nm) | Intensity: 0.0000% |
| Laser Line ( 488 nm) | Intensity: 0.0000% |
| Laser Line ( 496 nm) | Intensity: 1.0005% |
| Laser Line ( 514 nm) | Intensity: 0.0000% |
| Laser Line ( 561 nm) | Intensity: 0.0000% |
| Laser Line ( 633 nm) | Intensity: 0.0000% |

**Sequential Setting Nr.2**

| HyD 1 | Inactive, Gain: 0, Offset: 0 |
| --- | --- |
| PMT 2 | Inactive, Gain: 0, Offset: 0 |
| HyD 3 | Inactive, Gain: 0, Offset: 0 |
| PMT 4 | Active, Gain: 0, Offset: 0 |
| PMT Trans | Inactive, Gain: 0, Offset: 0 |
| Laser Line ( 405 nm) | Intensity: 0.0000% |
| Laser Line ( 458 nm) | Intensity: 0.0000% |
| Laser Line ( 476 nm) | Intensity: 0.0000% |
| Laser Line ( 488 nm) | Intensity: 0.0000% |
| Laser Line ( 496 nm) | Intensity: 0.0000% |
| Laser Line ( 514 nm) | Intensity: 0.0000% |
| Laser Line ( 561 nm) | Intensity: 0.0499% |
| Laser Line ( 633 nm) | Intensity: 0.0000% |

##

## **Supplementary file 2 - Liposomes**

**Dimensions**

| **Dimension** | **Logical Size** | **Physical Length** | **Start Position** | **End Position** | **Pixel Size / Voxel Size** |
| --- | --- | --- | --- | --- | --- |
| X | 512 | 144.72 µm | 0 µm | 144.72 µm | 0.283 µm |
| Y | 512 | 144.72 µm | 10.24 µm | 154.96 µm | 0.283 µm |

**Channels**

| **LUT** | **Resolution** | **Min** | **Max** | **STED: DetectorMode / Huygens saturation factor / Wavelength** |
| --- | --- | --- | --- | --- |
| Green 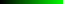 | 8 | 0 | 255 | --- / --- / --- |
| Red 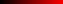 | 8 | 0 | 255 | --- / --- / --- |

**Time Stamps:**

| **Frame (Show All)** | **Relative Time (s)** | **Absolute Time (h:m:s.ms)** | **Date** |
| --- | --- | --- | --- |
| 1 | 0.000 | 10:24:35 AM.397 | 10/4/2022 |
| 2 | 0.000 | 10:24:35 AM.397 | 10/4/2022 |

**Confocal Settings**

| **Name** | **Value** |
| --- | --- |
| Rotator Angle | 0 ° |
| Scan Mode | xyz |
| Scan Direction X | Bidirectional |
| Scan Speed | 200 Hz |
| Version Number | 15 |
| StagePosX | 64,653.83 µm |
| StagePosY | 41,204.19 µm |
| ZPosition | -18.85 µm |
| IsSuperZ | 0 |
| Magnification | 63 |
| ObjectiveName | HC PL APO CS2 63x/1.20 WATER |
|  |  |

| Immersion | WATER |
| --- | --- |
| Numerical Aperture | 1.2 |
| RefractionIndex | 1.33 |
| Zoom | 1.28 |
| Pinhole | 83.5 µm |
| PinholeAiry | 749.52 mAU |
| EmissionWavelength for PinholeAiry Calculation | 580 nm |
| MotCorrPosition | 39.8 °% |
| FrameAverage | 1 |
| LineAverage | 2 |
| FrameAccumulation | 1 |
| Line_Accumulation | 1 |
| IsUserSettingNameSet | 0 |
| IsRoiScanEnable | 0 |

Filter Wheels / Other Motorized Devices

| **Device Name** | **Filter Name/Position** |
| --- | --- |
| Galvo Slider | Galvo X Normal |
| Galvo Resonant Pan | Galvo X Pan Center |
| Target Slider | Target Park |
| X2 Lens Changer | CS2 UV Optics 1 |

Lasers

| **LaserName** | **OutputPower** |
| --- | --- |
| **Diode 405** | **Off** |
| **Argon** | **On, 29.3651 %** |
| **DPSS 561** | **On** |
| **HeNe 633** | **Off** |

Laser Lines

| **Laser Line** | **Intensity** |
| --- | --- |
| ( 405 nm) | Shutter: off, Intensity: 0.0000% |
| ( 458 nm) | Shutter: on, Intensity: 0.0000% |
| ( 476 nm) | Shutter: on, Intensity: 0.0000% |
| ( 488 nm) | Shutter: on, Intensity: 0.0000% |
| ( 496 nm) | Shutter: on, Intensity: 1.0005% |
| ( 514 nm) | Shutter: on, Intensity: 0.0000% |
| ( 561 nm) | Shutter: on, Intensity: 0.0000% |
| ( 633 nm) | Shutter: on, Intensity: 0.0000% |

Detectors

| **Name** | **Channel** | **Type** | **Location** | **Active** | **Gain** | **Offset** | **Gate Start** | **Gate End** | **Gate Ref. Wavelength** |
| --- | --- | --- | --- | --- | --- | --- | --- | --- | --- |
| HyD 1 | Channel 1 | HyD (380nm - 399nm) Standard mode | Internal | Inactive | 100 | -- | -- Time Gating not supported -- | | |
| PMT 2 | Channel 2 | PMT (501nm - 560nm) | Internal | Inactive | 800 | 0 | -- Time Gating not supported -- | | |
| HyD 3 | Channel 3 | HyD (561nm - 566nm) Standard mode | Internal | Inactive | 100 | -- | -- Time Gating not supported -- | | |
| PMT 4 | Channel 4 | PMT (566nm - 688nm) | Internal | Active | 800 | 0 | -- Time Gating not supported -- | | |
| PMT Trans | Transmission Channel | PMT | TLD | Inactive | 0 | 0 | -- Time Gating not supported -- | | |

**Sequential Setting Nr.1**

| HyD 1 | Inactive, Gain: 0, Offset: 0 |
| --- | --- |
| PMT 2 | Active, Gain: 0, Offset: 0 |
| HyD 3 | Inactive, Gain: 0, Offset: 0 |
| PMT 4 | Inactive, Gain: 0, Offset: 0 |
| PMT Trans | Inactive, Gain: 0, Offset: 0 |
| Laser Line ( 405 nm) | Intensity: 0.0000% |
| Laser Line ( 458 nm) | Intensity: 0.0000% |
| Laser Line ( 476 nm) | Intensity: 0.0000% |
| Laser Line ( 488 nm) | Intensity: 0.0000% |
| Laser Line ( 496 nm) | Intensity: 1.0005% |
| Laser Line ( 514 nm) | Intensity: 0.0000% |
| Laser Line ( 561 nm) | Intensity: 0.0000% |
| Laser Line ( 633 nm) | Intensity: 0.0000% |

**Sequential Setting Nr.2**

| HyD 1 | Inactive, Gain: 0, Offset: 0 |
| --- | --- |
| PMT 2 | Inactive, Gain: 0, Offset: 0 |
| HyD 3 | Inactive, Gain: 0, Offset: 0 |
| PMT 4 | Active, Gain: 0, Offset: 0 |
| PMT Trans | Inactive, Gain: 0, Offset: 0 |
| Laser Line ( 405 nm) | Intensity: 0.0000% |
| Laser Line ( 458 nm) | Intensity: 0.0000% |
| Laser Line ( 476 nm) | Intensity: 0.0000% |
| Laser Line ( 488 nm) | Intensity: 0.0000% |
| Laser Line ( 496 nm) | Intensity: 0.0000% |
| Laser Line ( 514 nm) | Intensity: 0.0000% |
| Laser Line ( 561 nm) | Intensity: 1.0005% |
| Laser Line ( 633 nm) | Intensity: 0.0000% |

## **Supplementary file 3 – Nanoparticles in Liposomes**

**Dimensions**

| **Dimension** | **Logical Size** | **Physical Length** | **Start Position** | **End Position** | **Pixel Size / Voxel Size** |
| --- | --- | --- | --- | --- | --- |
| X | 512 | 42.52 µm | 0 µm | 42.52 µm | 0.083 µm |
| Y | 512 | 42.52 µm | 10.24 µm | 52.75 µm | 0.083 µm |
| Z | 27 | 7.59 µm | -23.84 µm | -16.25 µm | 0.292 µm |

**Channels**

| **LUT** | **Resolution** | **Min** | **Max** | **STED: DetectorMode / Huygens saturation factor / Wavelength** |
| --- | --- | --- | --- | --- |
| Green 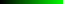 | 8 | 0 | 255 | --- / --- / --- |
| Red 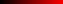 | 8 | 0 | 255 | --- / --- / --- |

**Time Stamps:**

| **Frame (Show All)** | **Relative Time (s)** | **Absolute Time (h:m:s.ms)** | **Date** |
| --- | --- | --- | --- |
| 1 | 0.000 | 10:00:01 AM.290 | 10/4/2022 |
| 54 | 134.415 | 10:02:15 AM.705 | 10/4/2022 |

**Confocal Settings**

| **Name** | **Value** |
| --- | --- |
| Rotator Angle | 0 ° |
| Scan Mode | xyz |
| Scan Direction X | Bidirectional |
| Scan Speed | 200 Hz |
| Version Number | 15 |
| StagePosX | 55,001.37 µm |
| StagePosY | 46,481.36 µm |
| ZPosition | -16.25 µm |
| IsSuperZ | 0 |
| Magnification | 63 |

| ObjectiveName | HC PL APO CS2 63x/1.20 WATER |
| --- | --- |
| Immersion | WATER |
| Numerical Aperture | 1.2 |
| RefractionIndex | 1.33 |
| Zoom | 4.34 |
| Pinhole | 83.5 µm |
| PinholeAiry | 749.52 mAU |
| EmissionWavelength for PinholeAiry Calculation | 580 nm |
| MotCorrPosition | 39.8 °% |
| FrameAverage | 1 |
| LineAverage | 2 |
| FrameAccumulation | 1 |
| Line_Accumulation | 1 |
| IsUserSettingNameSet | 0 |
| IsRoiScanEnable | 0 |

Filter Wheels / Other Motorized Devices

| **Device Name** | **Filter Name/Position** |
| --- | --- |
| Galvo Slider | Galvo X Normal |
| Galvo Resonant Pan | Galvo X Pan Center |
| Target Slider | Target Park |
| X2 Lens Changer | CS2 UV Optics 1 |

Lasers

| **LaserName** | **OutputPower** |
| --- | --- |
| **Diode 405** | **Off** |
| **Argon** | **On, 29.3651 %** |
| **DPSS 561** | **On** |
| **HeNe 633** | **Off** |

Laser Lines

| **Laser Line** | **Intensity** |
| --- | --- |
| ( 405 nm) | Shutter: off, Intensity: 0.0000% |
| ( 458 nm) | Shutter: on, Intensity: 0.0000% |
| ( 476 nm) | Shutter: on, Intensity: 0.0000% |
| ( 488 nm) | Shutter: on, Intensity: 0.0000% |
| ( 496 nm) | Shutter: on, Intensity: 1.0005% |
| ( 514 nm) | Shutter: on, Intensity: 0.0000% |
| ( 561 nm) | Shutter: on, Intensity: 0.0000% |
| ( 633 nm) | Shutter: on, Intensity: 0.0000% |

Detectors

| **Name** | **Channel** | **Type** | **Location** | **Active** | **Gain** | **Offset** | **Gate Start** | **Gate End** | **Gate Ref. Wavelength** |
| --- | --- | --- | --- | --- | --- | --- | --- | --- | --- |
| HyD 1 | Channel 1 | HyD (380nm - 399nm) Standard mode | Internal | Inactive | 100 | -- | -- Time Gating not supported -- | | |
| PMT 2 | Channel 2 | PMT (501nm - 560nm) | Internal | Inactive | 800 | 0 | -- Time Gating not supported -- | | |
| HyD 3 | Channel 3 | HyD (561nm - 566nm) Standard mode | Internal | Inactive | 100 | -- | -- Time Gating not supported -- | | |
| PMT 4 | Channel 4 | PMT (566nm - 688nm) | Internal | Active | 800 | 0 | -- Time Gating not supported -- | | |
| PMT Trans | Transmission Channel | PMT | TLD | Inactive | 0 | 0 | -- Time Gating not supported -- | | |

**Sequential Setting Nr.1**

| HyD 1 | Inactive, Gain: 0, Offset: 0 |
| --- | --- |
| PMT 2 | Active, Gain: 0, Offset: 0 |
| HyD 3 | Inactive, Gain: 0, Offset: 0 |
| PMT 4 | Inactive, Gain: 0, Offset: 0 |
| PMT Trans | Inactive, Gain: 0, Offset: 0 |
| Laser Line ( 405 nm) | Intensity: 0.0000% |
| Laser Line ( 458 nm) | Intensity: 0.0000% |
| Laser Line ( 476 nm) | Intensity: 0.0000% |
| Laser Line ( 488 nm) | Intensity: 0.0000% |
| Laser Line ( 496 nm) | Intensity: 1.0005% |
| Laser Line ( 514 nm) | Intensity: 0.0000% |
| Laser Line ( 561 nm) | Intensity: 0.0000% |
| Laser Line ( 633 nm) | Intensity: 0.0000% |

**Sequential Setting Nr.2**

| HyD 1 | Inactive, Gain: 0, Offset: 0 |
| --- | --- |
| PMT 2 | Inactive, Gain: 0, Offset: 0 |
| HyD 3 | Inactive, Gain: 0, Offset: 0 |
| PMT 4 | Active, Gain: 0, Offset: 0 |
| PMT Trans | Inactive, Gain: 0, Offset: 0 |
| Laser Line ( 405 nm) | Intensity: 0.0000% |
| Laser Line ( 458 nm) | Intensity: 0.0000% |
| Laser Line ( 476 nm) | Intensity: 0.0000% |
| Laser Line ( 488 nm) | Intensity: 0.0000% |
| Laser Line ( 496 nm) | Intensity: 0.0000% |
| Laser Line ( 514 nm) | Intensity: 0.0000% |
| Laser Line ( 561 nm) | Intensity: 1.0005% |
| Laser Line ( 633 nm) | Intensity: 0.0000% |

**METHODS:**

**Synthesis of 3C:** The synthesis of the experimental compound was accomplished via one step. An amount of 0.5 g (3.99 mmol) of 2-amino-5-cyanothiazole was added to 1.125 g (5.99 mmol) of 3, 4-dichloro phenyl isocyanate. The reaction was done in 10 ml Dimethylformamide (DMF) as solvent. The mixture was left to stir at room temperature overnight, and afterwards, the solvent was evaporated under vacuo and the product was purified by silica gel column chromatography. The column chromatography was performed using Hexane/Ethyl Acetate 1:1.

**Development of Ultra-high performance liquid chromatography- tandem mass spectrometer (UHPLC-MS/MS) method for quantification of compounds:**

**3C:**

UHPLC-MS/MS method for determination of 3C was performed using diphenhydramine as internal standard. Analyses were acquired on a Waters ACQUITY Xevo TQD system, which consisted of ACQUITY UPLC H-Class system and XevoTQD triplequadrupole tandem mass spectrometer with an electrospray ionization (ESI) interface (Waters Corp., Milford, MA, USA). Aquity UPLC BEH C18 50 mm × 2.1 mm column (particle size, 1.7 µm) was used to separate analytes (Waters, Wexford, Ireland). System operation and data acquisition were controlled using MassLynx 4.1 software (Waters, Milford, MA, USA). All data were processed with the TargetLynx quantification program (Waters, Milford, MA, USA). A gradient elution at a flow rate of 0.2 ml/minute was conducted for chromatographic separation using 0.1 % formic acid in water (A) and 0.1 % formic acid in acetonitrile (B). The gradient was run as follows: 0 minutes, 95 % A, 5 % B; 1.0-minute, 5 % A, 95 % B; 3 minutes, 5 % A, 95 % B; 4 minutes, 95 % A, 5 % B; 6 minutes, 95 % A, 5 % B. The injection volume was 10 µl performed by partial loop injection using needle overfill as injection technique. The column temperature was set at 40 ºC. Nitrogen was used as the desolvation and cone gas at a flow rate of 1000 and 1 L/hour, respectively. Argon was used as the collision gas at a pressure of approximately 3.67 × 10−3 mbar. The optimal MS parameters were as follows: capillary voltage 3.9 kV, radio frequency (RF) lens voltage 2.5V, source temperature 150 ºC and desolvation gas temperature 500 ºC. Cone voltage was 32 and 38V for 3C and diphenhydramine, respectively. The ESI source was operated in positive mode. Quantification was performed using multiple reaction monitoring (MRM) of the transitions of m/z 312.96>126.04 with collision energy of 18V for 3C and m/z 256.14>167.04 with collision energy of 10V for diphenhydramine respectively. Dwell time was automatically set by Mass Lynx 4.1 software.

**HIPS-1635:**

The UHPLC-MS/MS method for determination of HIPS-1635 was performed using diphenhydramine as internal standard. Analyses were acquired on a Waters ACQUITY Xevo TQD system, which consisted of ACQUITY UPLC H-Class system and XevoTQD triple-quadrupole tandem mass spectrometer with an electrospray ionization (ESI) interface (Waters Corp., Milford, MA, USA). Aquity UPLC BEH C_18_ 100 mm × 2.1 mm column (particle size, 1.7 µm) was used to separate analytes (Waters, Wexford, Ireland). System operation and data acquisition were controlled using MassLynx 4.1 software (Waters, Milford, MA, USA). All data were processed with the TargetLynx quantification program (Waters, Milford, MA, USA). A gradient elution at a flow rate of 0.2 mL/min was conducted for chromatographic separation using 0.1% formic acid in water (A) and 0.1% formic acid in acetonitrile (B). The gradient was run as follows: 0 min, 95% A, 5% B; 1.0 min, 5% A, 95% B; 3 min, 5% A, 95% B; 4 min, 95 % A, 5% B; 6 min, 95 % A, 5% B. The injection volume was 10 µL performed by partial loop injection using needle overfill as injection technique. The column temperature was set at 40 ^o^C. Nitrogen was used as the desolvation and cone gas at a flow rate of 1000 and 1 L/h, respectively. Argon was used as the collision gas at a pressure of approximately 3.67 × 10^−3^ mbar. The optimal MS parameters were as follows: capillary voltage 3.9 kV, radio frequency (RF) lens voltage 2.5V, source temperature 150 ^o^C and desolvation gas temperature 500 ^o^C. Cone voltage was 48 and 23V for HIPS-1635 and diphenhydramine respectively. The ESI source was operated in positive mode. Quantification was performed using multiple reaction monitoring (MRM) of the transitions of m/z 423.07>191.94 with collision energy of 24V for HIPS-1635 and m/z 256.14>167.04 with collision energy of 10V for diphenhydramine respectively. Dwell time was automatically set by MassLynx 4.1 software.

**Tobramycin**

For liquid chromatography- mass spectrometer (LC-MS) analysis of Tobramycin (Tbr), a Dionex UltiMate 3000 Binary Rapid Separation LC System (Thermo Scientific, USA) coupled with a TSQ Quantum Access Max (QQQ, Thermo Scientific, USA) was used. The analytical column is a Zorbax Eclipse xdb C-18 column (5 µm, 50*4,6 mm, Agilent, USA) with a C18 guard column. As mobile phase acetonitrile (eluent A) and water (eluent B), each supplemented with 0.1 % trifluoracetic acid, 0.1% heptafluorobutyric acid and 0.1% pentafluoropropionic acid were used. Samples were run with a flow of 0.7 mL/min, using a gradient of eluents A and B, starting with a ratio of 20:80 in the first minute. From 1 to 3.5 min the ratio was changed to 70:30 and was restored to 20:80 between 3.5 and 4.5 min. 5 μL of the samples was injected. The measurement was done doing a SRM (selected reaction monitoring) in positve ion mode using a H-ESI Probe for Ionization with the ion transition of 468.184 à 323.960 m/z.

**
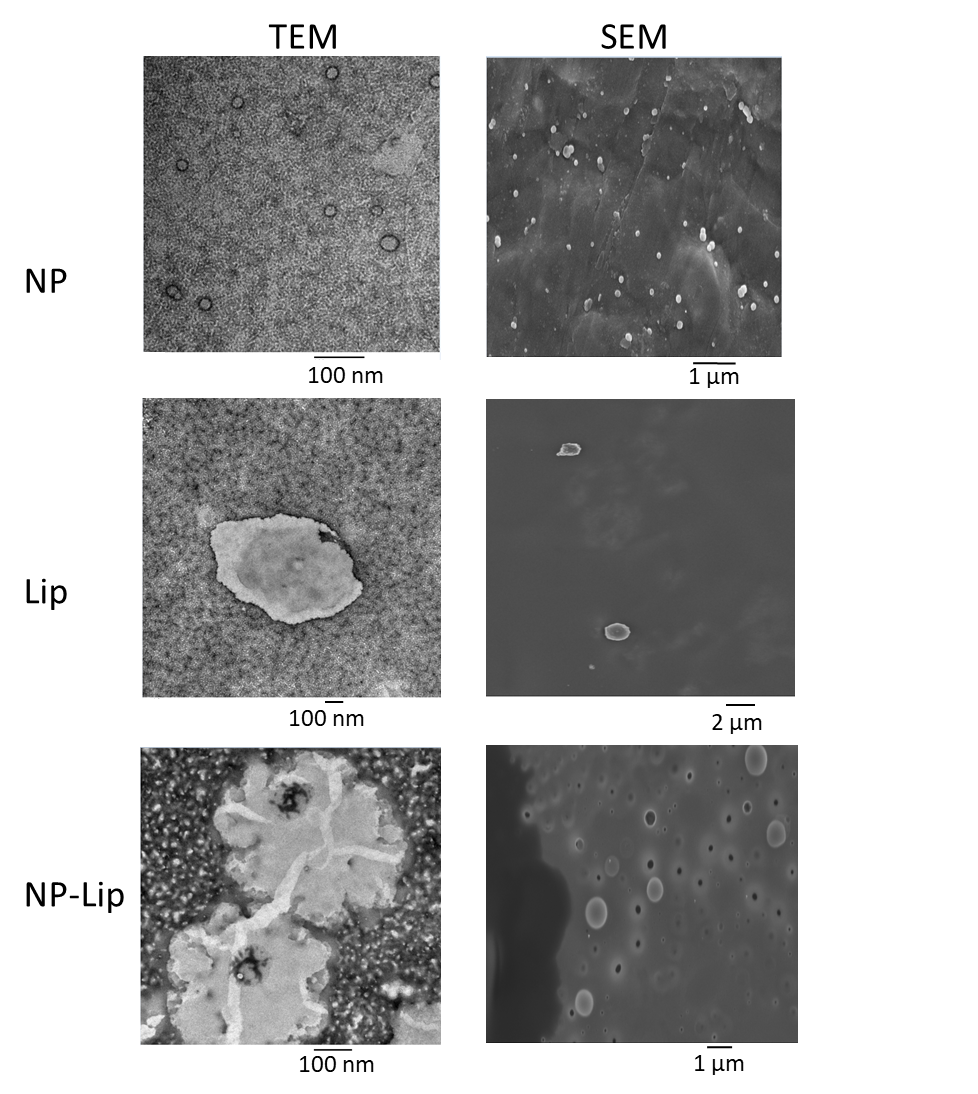
**

**Figure A:** TEM and SEM images of NP, Lip and NP-Lip. For TEM images, NP images were captured at magnification of 80,000, Lip images were captured at a magnification of 30,000 and NP-Lip images were captured at a magnification of 60,000. For SEM images, NP and NP-Lip images were captured at a magnification of 10,000, while Lip images were captured at a magnification of 2000.

**Table A: Treatment groups for *P. aeruginosa* biofilms and Calu-3 cells and their concentrations presented as final concentration per well**

| **Formulations tested** | **Concentration** | | | | | |
| --- | --- | --- | --- | --- | --- | --- |
| 3C (μg/ml) | 2.5 | 5.0 |  |  |  |  |
| Tbr (μg/ml) | 4.7 | 10.0 |  |  |  |  |
| HIPS-1635 (μM) | 20 | 50 | 100 |  |  |  |
| 3C (μg/ml) +  HIPS-1635 (μM) | 2.5 | 2.5 | 2.5 | 5.0 | 5.0 | 5.0 |
|  | 20 | 50 | 100 | 20 | 50 | 100 |
| Tbr (μg/ml) +  HIPS-1635(μM) | 4.7 | 4.7 | 4.7 | 10.0 | 10.0 | 10.0 |
|  | 20 | 50 | 100 | 20 | 50 | 100 |
| 3C NPs (μg/ml) | 2.5 | 5.0 |  |  |  |  |
| 3C NPs (μg/ml) +  HIPS-1635 (μM) | 2.5 | 2.5 | 2.5 | 5.0 | 5.0 | 5.0 |
|  | 20 | 50 | 100 | 20 | 50 | 100 |
| 3C NP-Lip (μg/ml) | 2.5 | 5.0 |  |  |  |  |
| 3C NP-Lip (μg/ml) +  HIPS-1635 (μM) | 2.5 | 2.5 | 2.5 | 5.0 | 5.0 | 5.0 |
|  | 20 | 50 | 100 | 20 | 50 | 100 |
| 3C NP (μg/ml)-  H1635-Lip (μM) | 2.5 | 5.0 |  |  |  |  |
|  | 87 | 176 |  |  |  |  |
| Tbr NPs (μg/ml) | 4.7 | 10.0 |  |  |  |  |
| Tbr NPs (μg/ml)+  HIPS-1635 (μM) | 4.7 | 4.7 | 4.7 | 10.0 | 10.0 | 10.0 |
|  | 20 | 50 | 100 | 20 | 50 | 100 |
| Tbr NP- Lip (μg/ml) | 4.7 | 10.0 |  |  |  |  |
| Tbr NP (μg/ml) -  H1635- Lip (μM) | 4.7 | 10.0 |  |  |  |  |
|  | 87 | 187 |  |  |  |  |
| NP-H1635-Lip indicates the encapsulation of HIPS-1635 in liposomes  NP-Lip +HIPS-1635 indicated co-treatment with HIPS-1635 and NP-Lip | | | | | | |


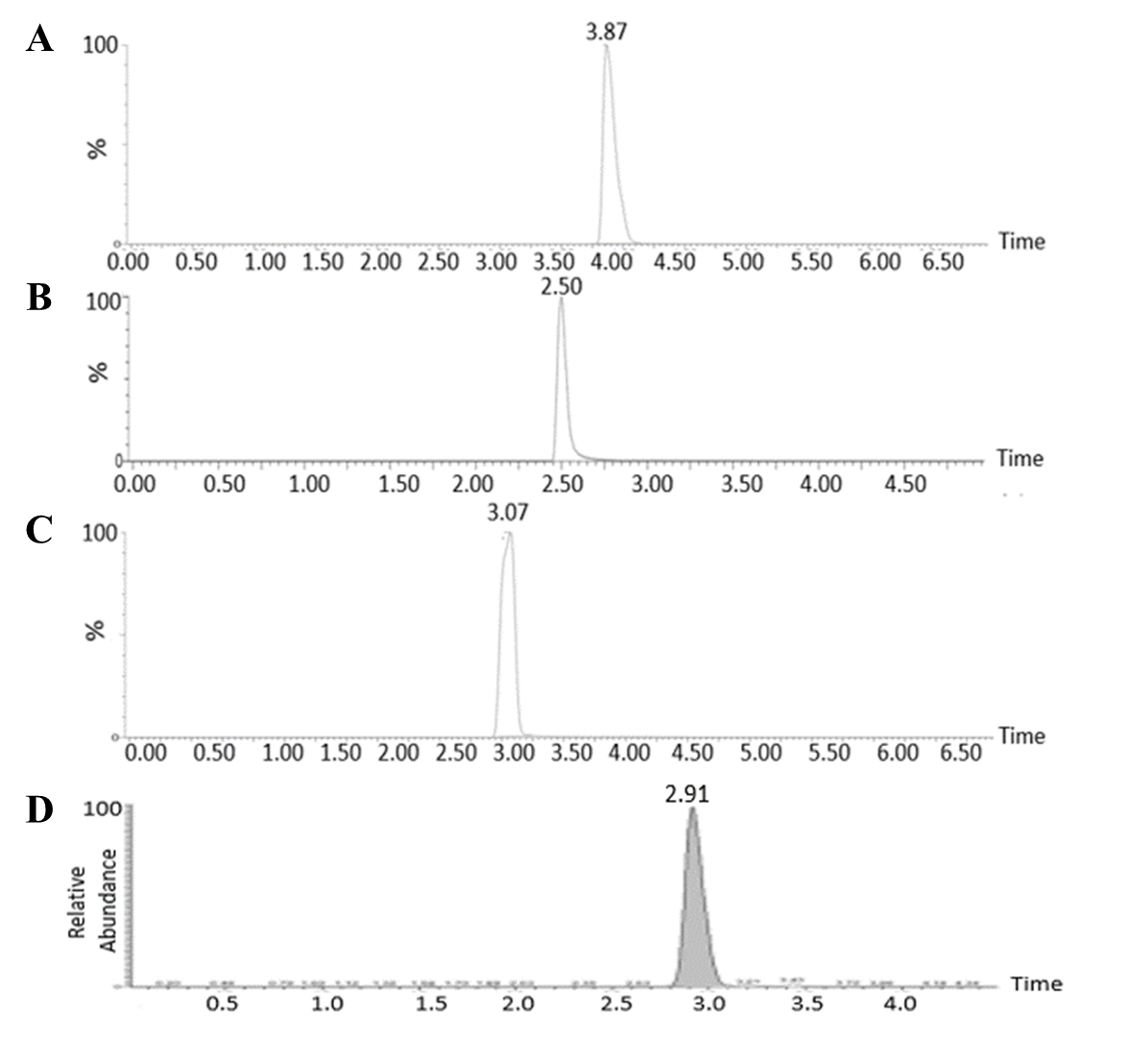


**E**

**F**

**G**

**Figure B:** LC-MS chromatograms of **(A)** 3C, **(B)** HIPS-1635, **(C)** their internal standard diphenhydramine and **(D)**Tbr. The calibration curves of **(E)** 3C, **(F)** HIPS-1635 and **(G)** Tbr.
